# The Cognitive Footprint of Medication Use

**DOI:** 10.1002/brb3.70200

**Published:** 2025-01-19

**Authors:** Marta Suárez Pinilla, Charlotte R. Stoner, Martin Knapp, Parashkev Nachev, Martin Rossor

**Affiliations:** ^1^ Clinical Neuroscience Laboratory, Center for Biomedical Technology Universidad Politécnica de Madrid Madrid Spain; ^2^ UCL Queen Square Institute of Neurology University College London London UK; ^3^ Centre for Chronic Illness and Ageing, School of Human Sciences University of Greenwich Park Row London; ^4^ Health Policy Department London School of Economics and Political Science London UK

**Keywords:** medications | cognitive footprint | large‐scale cohorts | medication side effects | cognition

## Abstract

**Introduction:**

The cognitive side‐effects of medication are common, but often overlooked in practice, and not routinely considered in interventional trials or post‐market surveillance. The cognitive footprint of a medication seeks to quantify the impact of its cognitive effects based on magnitude, duration, and interaction with other factors, evaluated across the exposed population.

**Methods:**

Bayesian multivariable regression analysis of retrospective population‐based cross‐sectional cohorts.

**Results:**

We replicate positive and negative cognitive effects of commonly used medications in UK Biobank, and extend observed associations to two additional cohorts, the EPIC Norfolk, and the Caerphilly Prospective Cohort. We quantify the resultant cumulative impact at the population level given known patterns of prescribing and compare it with exemplar common diseases.

**Conclusion:**

The cognitive side‐effects of commonly used drugs may have significant impact at the population level. Consideration should be given to a routine structured assessment of cognition in interventional trials and post‐market surveillance.

## Graphical abstract

The cognitive footprint of medication use is defined as the positive or negative effect of the regular consumption of different medications on the cognitive resources of the overall population). Cognitive footprint was explored on three large population cohorts in the United Kingdom: UK Biobank (over 500,000 participants), EPIC Norfolk and CaPS. The figure shows positive and negative associations observed on the UK Biobank cohort on overall cognition for a wide array of medications; the effect, expressed in standardized units, is the result of applying the model estimates to the prevalence of medication use in the UK population. Some medications or dietary supplements have a positive footprint, such as ibuprofen and glucosamine, whereas others have a negative footprint, most notably paracetamol. For comparison, the population‐based footprint of other factors is also presented, showing that the cognitive effect of some medications is of comparable magnitude to chronic pain, mental disorders related to substance abuse or air pollution.

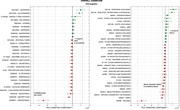

## 1 Introduction

Cognition is plausibly influenced by multiple modifiable factors that can be targeted by public policy interventions, with the dual aims of maximizing cognitive resources and reducing inequalities across the population(Beddington et al. 2008; Bradley and Corwyn 2002; Capron and Duyme 1989; Clifford et al. 2016; Clouston et al. 2012; Duncan and Magnusson 2012; Falck et al. 2017; Fan and Wong 2019; Farah 2017; Hanushek and Woessmann 2008; Huang et al. 2012; Hunt et al. 2020; Lees and Hopkins 2013; Lehert et al. 2015; Lupien et al. 2009; Phillips C. 2017; Topiwala and Ebmeier 2018). To identify safe and effective interventions, it is important to ascertain the impact that each potentially modifiable factor has on cognition at a societal level. The ‘cognitive footprint’ of a considered factor aims to quantify short and long term effects by estimating both the magnitude and duration of effects on cognition, with sensitivity to the clinical, sociodemographic and cultural contexts (Rossor and Knapp 2015; Cullen et al. 2017).

The impact of medication has been relatively under‐investigated, especially for medications that do not primarily target the central nervous system (CNS) (Nevado‐Holgado et al. 2016). Medication use is especially frequent in older people (Payne et al. 2014; Moriarty et al. 2015), where chronic disorders and multiple long‐term conditions combine with heightened sensitivity to drug side‐effects. The prevalence of older adults in the United States prescribed at least three medications with established negative effects on cognition increased by 300% between 2000 and 2016; furthermore, the use of these medications was associated with significant impairment on cognitive tests in a nationwide representative sample (Do and Schnittker 2020). Those authors conjectured that cognitive effects receive little attention because of misattribution to age‐related decline. In addition to prescription medications, use of over‐the‐counter products and dietary supplements is common, often without adequate medical justification (Fulton and Allen 2005; Payne 2016). Conversely, some medications may have a positive effect on cognition, either as temporary enhancers of performance (Husain and Mehta 2011), or in primary prevention or modification of the course of neurodegeneration (Corbett, Williams, and Ballard 2013). Even a small effect size may have significant impact at the population level if the medication is taken by many people or for a long time.

In this study we aimed to extend the analysis of Nevado‐Holgado et al. (2016) of medication use in UK Biobank within a Bayesian modeling framework, facilitating the interpretation of null findings and quantification of population‐level effects, and to validate our results in two other smaller cohorts, EPIC Norfolk and Caerphilly Prospective Cohort (CaPS). We employed a cognitive footprint framework, accounting for sociodemographic variables, medical history and relevant modifiable factors of cognition, and considering the overall impact in relation to the frequency of use of each medication in a naturalistic sample.

## 2 Methods

UK Biobank (Allen et al. 2012; Sudlow et al. 2015) is a UK‐wide, ongoing observational cohort composed of 502,492 participants recruited between April 2007 and July 2010, with baseline ages ranging from 37 to 73 years (mean 56.5). Data were collected at baseline (instance 0, 2007–2010) and in three subsequent follow‐up visits. Cognitive variables are summarized in Table 2.

EPIC Norfolk (Hayat et al. 2014), part of the wider European Prospective Investigation of Cancer, recruited 25,639 participants aged between 40 and 79 years at enrolment. Data were collected in five waves, but cognitive tests were performed only in wave 3 (2004–2011), on a subset of 8623 participants.

The Caerphilly Prospective Study (CaPS) (“Caerphilly, and Speedwell collaborative heart disease studies. The Caerphilly, and Speedwell Collaborative Group,” 1984) enrolled 2959 male volunteers between 45–59 years old between 1979 and 1983, with five data‐collecting waves in total; cognitive assessments were performed from wave 3 (1989–1993) to wave 5 (2002–2004).

Cognitive performance was measured by a different set of tests for each of the three cohorts, with only partial overlap (Table 1). All numeric variables were normalized, and sign reversed if needed, so that a larger score consistently indicated better performance.

**TABLE 1:** Description of cognitive variables in the three cohorts (UK Biobank, EPIC Norfolk, CaPS), indicating the cognitive tests from which they are produce and the cognitive domains probed by those tests. Cells shaded in grey correspond to cognitive tests that were run for the first time in the second follow‐up visit (instance 2) of the UK Biobank cohort, on a much smaller sample than baseline tests: Analyses for these tests are reported in the Supporting Information.

Cognitive domain	UK Biobank	EPIC Norfolk	CaPS
Speed of processing	**RT** (reaction time): mean time to correctly identify if a pair of cards was matched.	**Simple processing speed**: **Simple‐VST**: mean reaction time of the simple visual sensitivity test.	**CRT** (choice reaction time): decision speed task.
		**Complex processing speed (and visual deficits)**: **Complex‐VST**: mean reaction time of the complex visual sensitivity test.	
Verbal/numerical reasoning	**FI**: fluid intelligence score (13 questions of verbal/numerical reasoning).		**AH4** ‐ Part 1: Alice Heim Group ability test. Part 1 is formed by 65 questions assessing verbal and mathematical reasoning.
Abstract nonverbal reasoning	**MPC**: matrix pattern completion (select an element that best fits an incomplete pattern).		
Premorbid IQ		**NART**: National adult reading test.	**NART**: National adult reading test.
Global cognitive function—detection of cognitive impairment		**SF‐EMSE**: Short form of the extended mental state Exam.	**CAMCOG**: Cambridge cognitive Examination. Clinical test covering a broad range of cognitive skills and functions.
		**MMSE**: Mini‐mental state exam.	**MMSE**: Mini‐Mental State Exam.
Memory	**Verbal memory** **PWL** (paired words associated learning).	**Verbal episodic memory** **HVLT**: Hopkins verbal learning test.	**Immediate recall** **IMM‐R** Rivermead behavioral memory test: recalling prose details.
			**Delayed recall** **DEL‐R** Rivermead behavioral memory test: recalling prose details.
			**Prompted recall** **PROMPT‐R** Rivermead behavioral memory test: recalling prose details.
			**Incidental memory** **INCID‐M**: recall a list of names that were read in another context.
	**Short‐term visual memory** **PaMa** (errors in a pairs‐matching test).	**Non‐verbal episodic memory** **CANTAB‐PAL**: paired associate learning test from the Cambridge Neuropsychological Test Battery.	
	**Numeric memory** **NM** (maximum number of digits recalled in a sequence).		
	**Prospective memory** **PM** (correct recall of an earlier instruction).	**Prospective memory** **PM** (correct recall of an earlier instruction).	**Prospective memory** **PM** (correct recall of an earlier instruction).
Attention		**LCT‐ACC**: Accuracy score in the letter cancellation task (MRC‐CFAS).	
Visual processing	**SDS**: symbol‐digit substitution (linking symbols and digits according to a provided key).		
Strategic planning	**TR**: tower rearranging (re‐arrange hoops around a peg to form a different color pattern in the least number of steps).		
Cognitive flexibility/executive functioning	**TMT**: trail‐making test (sequentially follow a trail).		

Cognition is a multi‐faced ability that nonetheless shows strong correlations across distinct facets. For the UK Biobank cohort, the first component of a principal component analysis (PCA) of different cognitive tests was therefore employed as a summary measure for underlying cognition (PCA‐cognition), in addition to models of each individual test. Further detail on all variables and operations performed on them before inclusion in the models is reported in the Supporting Information.

Medications are naturally heterogeneous but exhibit commonalities arising from shared chemical and biological properties that permit therapeutic domain‐level analysis. All medications were coded according to the last two levels of the anatomical therapeutic chemical (ATC) classification system, i.e., level 5 (chemical substances, e.g., M01AE01: ibuprofen) and 4 (pharmacological subgroups, e.g., M01AE: propionic acid non‐steroidal anti‐inflammatory drugs). The association between medication and cognition was modeled separately for these two descriptive levels.

Isolating the specific effect of an intervention demands modeling of collateral factors with a bearing on cognition. In modeling the association between medications (as predictors) and cognitive performance (as the dependent variable), other covariates such as socio‐demographic information and medical history were considered, selected by a priori biological plausibility, previous evidence, data availability, and exploratory modeling. The specific variables included in the models varied for each of the three cohorts, depending on availability of the variables themselves, missing data, and constraints related to the number of predictors, which varied as a function of the sample size and the type of model employed. Particularly, UK‐Biobank analyses were highly comprehensive, as allowed by the large size of the cohort and the use of Bayesian penalized regression models, which are suitable for modeling a very large number of potential predictors in exploratory settings where most are likely uncorrelated with the outcome. Model predictors included socio‐demographic information (age, sex, ethnicity, household income, academic attainment, professional status, deprivation index and environmental conditions associated to residential address), lifestyle (alcohol and other toxics, physical activity), medical history (including every past and present diagnosis with over 1% prevalence in the UK‐Biobank sample), quantitative measures related to metabolic and cardiovascular health (body mass index, blood pressure, glycosylated hemoglobin), menopausal status in women, general physical and psychological welfare (self‐perceived health, mood, pain) and every medication with over 1% prevalence of regular use in the sample. EPIC and CaPS were smaller and less exhaustive, so the models were also less detailed, but aimed to resemble the UK‐Biobank models as far as possible. Cross‐sectional analysis (UK‐Biobank and EPIC) was carried out by high‐dimensional Bayesian penalized linear (or logistic) regression with sparse horseshoe‐shaped priors while repeated‐measures analysis was performed by means of mixed‐effects modeling (CaPS). The overall cognitive footprint of each medication in the UK population according to the results from the UK Biobank cohort was calculated assuming that the prevalence of medication use, stratified by age and sex, was the same as within UK Biobank.

## 3 Results

### 3.1 UK Biobank

Table 2 shows the available sample size and selected descriptive statistics for the data involved in each model. Table 3 presents the coefficient estimates and 95% credible intervals for the 10 medications that were more strongly associated with each cognitive measure, according to their rank statistic. All cognitive variables were normalized; thus, the value of the coefficients is comparable across models (except for the dichotomous prospective memory task (PM)).

**TABLE 2:** Sample size and descriptive statistics for selected variables in the cross‐sectional models for cognition. Regarding medical conditions and medications (ATC level 5), only those in at least 5% of at least one model sample are detailed in this table, although all of those with a frequency over 1% were included as variables. The differences between models are explained by different availability of results for each cognitive test, as not all Biobank participants undertook all tests.

		UK Biobank: cognitive tests					
		FI	NM	RT	PaMa	PM	PCA C1
N		148,857	46,531	417,765	408,094	153,705	146,985
Cognitive score (before normalization)		5.98 (2.17)	6.54 (1.79)	559.48 (117.97)	4.77 (3.72)	76.56%	0.03 (1.25)
Age		56.74 (8.14)	56.55 (8.25)	56.57 (8.08)	56.49 (8.08)	56.81 (8.15)	56.71 (8.14)
Sex	Female	54.23%	54.45%	54.18%	54.27%	54.19%	54.29%
Ethnicity	White (any)	92.93%	95.42%	94.85%	95.19%	91.97%	93.14%
Household income (GBP)	<18,000	18.69%	19.70%	19.26%	19.03%	19.14%	18.56%
	<31,000	22.10%	22.76%	21.98%	22.07%	21.92%	22.12%
	<52,000	22.75%	23.10%	22.51%	22.76%	22.33%	22.84%
	<100,000	18.09%	17.00%	17.51%	17.75%	17.65%	18.17%
	>100,000	5.17%	3.86%	4.65%	4.71%	5.04%	5.20%
	N/A	13.20%	13.59%	14.07%	13.69%	13.92%	13.10
Highest academic qualification	Never schooled	0.05%	0.07%	0.06%	0.05%	0.08%	0.04%
	None	26.83%	28.74%	29.22%	28.69%	27.90%	26.69%
	CSE	5.76%	6.05%	5.76%	5.81%	5.74%	5.78%
	O levels	21.69%	22.53%	21.79%	21.96%	21.40%	21.74%
	A levels	11.59%	11.20%	11.19%	11.28%	11.40%	11.61%
	University	34.09%	31.41%	31.99%	32.22%	33.48%	34.13%
Professional qualifications	Yes	43.30%	44.57%	42.73%	43.06%	42.66%	43.44%
Alcohol drinker status	Current	92.07%	92.01%	92.26%	92.53%	91.47%	92.16%
	Previous	3.58%	3.78%	3.50%	3.45%	3.63%	3.57%
	Never	4.27%	4.13%	4.14%	3.93%	4.76%	4.19%
Hypertension and hypertensive diseases		10.12%	10.19%	9.72%	9.57%	10.38%	10.08%
Dyslipidemia		15.82%	15.09%	15.00%	14.85%	16.10%	15.79%
Hypothyroidism		5.50%	5.78%	5.25%	5.24%	5.54%	5.49%
Ischemic heart disease		5.24%	5.39%	5.25%	5.16%	5.39%	5.21%
Cancer		7.84%	7.62%	7.66%	7.64%	7.83%	7.82%
Rhinitis		8.07%	9.18%	8.74%	8.77%	8.00%	8.08%
Asthma		11.60%	11.38%	11.79%	11.79%	11.60%	11.61%
Inflammatory polyarthropaties		7.14%	6.90%	5.83%	5.78%	7.21%	7.13%
Osteoarthritis/arthrosis		12.06%	13.38%	12.87%	12.78%	12.20%	12.06%
Migraine		3.57%	3.81%	4.08%	4.09%	3.55%	3.47%
Major depression		8.20%	8.99%	8.21%	8.17%	8.25%	8.20%
Anxious mood		54.38%	54.13%	54.74%	54.67%	54.43%	54.35%
Depressed mood in the last 2 weeks	Any frequency	22.83%	22.39%	22.37%	22.31%	22.98%	22.81%
Invalidating pain last month		57.68%	58.27%	60.39%	60.27%	58.07%	57.65%
A02BC01 (omeprazole)		5.96%	6.09%	5.66%	5.61%	6.08%	5.95%
A11CA01 (vit A)		3.39%	2.74%	5.59%	5.58%	3.37%	3.40%
A11CC05 (vit D)		5.97%	4.88%	7.95%	7.91%	6.03%	5.96%
B01AC06 (aspirin)		12.19%	12.99%	13.65%	13.51%	12.43%	12.14%
C03AA01 (bendroflumethiazide)		5.76%	5.94%	5.96%	5.91%	5.85%	5.73%
C08CA01 (amlodipine)		5.74%	5.45%	5.15%	5.08%	5.89%	5.71%
C09AA05 (ramipril)		5.26%	4.85%	4.71%	4.66%	5.33%	5.24%
C10AA01 (simvastatin)		12.46%	12.31%	11.55%	11.44%	12.68%	12.41%
C10AA01 (atorvastatin)		3.41%	2.90%	3.64%	3.61%	3.47%	3.41%
C10AX06 (omega 3)		5.71%	5.28%	9.04%	9.05%	5.66%	5.73%
H03AA01 (L‐thyroxine)		5.82%	6.12%	5.60%	5.58%	5.87%	5.82%
M01AE01 (ibuprofen)		12.55%	13.38%	12.44%	12.50%	12.4%	12.60%
M01AX05 (glucosamine)		4.54%	4.50%	6.53%	6.55%	4.49%	4.54%
N02BE01 (paracetamol)		19.91%	22.89%	20.71%	20.59%	20.18%	19.88%
R03AC02 (salbutamol)		5.08%	5.37%	5.24%	5.23%	5.10%	5.09%

**TABLE 3:** presents the coefficient estimates and 95% credible intervals for the 10 medications that were more strongly associated with each cognitive measure in the UK Biobank cohort, according to their rank statistic. Medications are coded according to the anatomical therapeutic chemical classification (ATC), levels 5 (chemical substances, left) and 4 (pharmacological subgroups, right). All cognitive variables were normalized; thus, the value of the coefficients is comparable across models (except for the dichotomous prospective memory task (PM)) positive coefficients indicate association to better cognitive performance and vice versa.

UK Biobank: medications most strongly associated with cognitive tests			
RT (speed of processing)			
ATC level 5 (*R* ^2^= 0.1376)		ATC level 4 (*R* ^2^= 0.1375)	
N03AG01 (valproic)	−0.20927 (−0.26827, −0.14946)*	N03AG (fatty acid derived antiepileptics)	−0.20957 (−0.26878, −0.15248)*
N02BE01 (paracetamol)	−0.02590 (−0.03403, −0.01765)*	N02BE (anilides)	−0.02657 (−0.03451, −0.01844)*
M01AE01 (ibuprofen)	0.02690 (0.01766, 0.03612)*	M01AE (propionic acid derived NSAIDs)	0.02811 (0.01917, 0.03704)*
A10A (insulin)	−0.08406 (−0.12348, −0.04361)*	A10A (insulin)	−0.08462 (−0.12431, −0.04594)*
N06AB06 (sertraline)	−0.12732 (−0.17376, −0.08000)*	N06AB (SSRIs)	−0.04276 (−0.05996, −0.02535)*
N06AB03 (fluoxetine)	−0.07203 (−0.09942, −0.04438)*	N06AA (nsMRIs)	−0.04744 (−0.06699, −0.02799)*
M01AX05 (glucosamine)	0.02596 (0.01158, 0.04010)*	G04BD (drugs for urinary frequency and incontinence)	−0.07713 (−0.10823, −0.04454)*
D02AX (emolients)	−0.08950 (−0.13176, −0.04679)*	M01AX (other antiinflammatory and antirheumatic)	0.02926 (0.01587, 0.04233)*
N05BA01 (diazepam)	−0.12445 (−0.18410, −0.06456)*	N05BA (benzodiazepine anxiolytics)	−0.13475 (−0.18998, −0.07940)*
N06AA09 (amitriptyline)	−0.03764 (−0.05997, −0.01460)*	N03AX (other antiepileptics)	−0.06659 (−0.09824, −0.03487)*
**FI (verbal/numerical reasoning)**			
**ATC level 5 (*R* ^2^= 0.2622)**		**ATC level 4 (*R* ^2^= 0.2624)**	
N02BE01 (paracetamol)	−0.05241 (−0.06539, −0.03990)*	N02BE (anilides)	−0.05283 (−0.06561, −0.04052)*
N06AA09 (amitriptyline)	−0.05993 (−0.09534, −0.02337)*	G03AC (progestogens)	0.09628 (0.05310, 0.13186)*
G03CA57 (conjugated estrogens)	−0.07913 (−0.13086, −0.02408)*	N03AX (other antiepileptics)	−0.09353 (−0.13961, −0.04431)*
M01AX05 (glucosamine)	0.03243 (0.00456, 0.05822)*	G03CA (estrogens)	−0.04883 (−0.07799, −0.01946)*
A02BC03 (lansoprazole)	−0.02926 (−0.05437, −0.00313)*	M01AX (other antiinflammatory and antirheumatic)	0.03958 (0.01434, 0.06331)*
M01AE01 (ibuprofen)	0.01331 (−0.00036, 0.02855)	A02BC (PPIs)	−0.02533 (−0.04144, −0.00860)*
G03CA03 (estradiol)	−0.02651 (−0.05951, 0.00143)	M01AE (propionic acid derived NSAIDs)	0.01830 (0.00283, 0.03269)*
M01AE02 (naproxen)	0.04112 (−0.00329, 0.09323)	N06AA (nsMRIs)	−0.04065 (−0.07211, −0.00600)*
R06AE07 (cetirizine)	0.03183 (−0.00260, 0.07344)	L04AX (other immunosuppressants)	0.05311 (−0.00152, 0.11352)
R03AC13 (formoterol)	−0.04763 (−0.12341, 0.00776)	G03DC (estren derivatives)	0.04811 (−0.00279, 0.10721)
**PaMa (short‐term visual memory)**			
**ATC level 5 (*R* ^2^= 0.0621)**		**ATC level 4 (*R* ^2^= 0.0621)**	
N02BE01 (paracetamol)	−0.02349 (−0.03188, −0.01477)*	N02BE (anilides)	−0.02422 (−0.03270, −0.01536)*
N05CF01 (zopiclone)	−0.10183 (−0.15358, −0.04927)*	N05BA (benzodiazepine anxiolytics)	−0.13274 (−0.19198, −0.07334)*
N06AB05 (paroxetine)	−0.08700 (−0.13555, −0.03751)*	N06AA (nsMRIs)	−0.04482 (−0.06548, −0.02408)*
N06AA09 (amitriptyline)	−0.04026 (−0.06460, −0.01546)*	N05CF (benzodiazepine hypnotics)	−0.10228 (−0.14911, −0.05549)*
A07EC01 (sulfasalazine)	−0.08760 (−0.14781, −0.02377)*	G03CA (estrogens)	−0.03633 (−0.05470, −0.01780)*
M01AX05 (glucosamine)	0.01885 (0.00193, 0.03428)*	M01AX (other antiinflammatory and antirheumatic)	0.02577 (0.01218, 0.03893)*
D11AX02 (gamolenic)	−0.03287 (−0.05787, −0.00605)*	N03AX (other antiepileptics)	−0.06371 (−0.09752, −0.02938)*
R06AB02 (dexchlorpheniramine)	0.08513 (0.01716, 0.14737)*	A03AX (drugs for functional gastrointestinal disorders)	−0.10747 (−0.16817, −0.04495)*
C08CA05 (nifedipine)	−0.05218 (−0.09413, −0.00788)*	M05BA (biphosphonates)	−0.03898 (−0.06488, −0.01151)*
G04BD08 (solifenacin)	−0.07167 (−0.13442, −0.00697)*	C08CA (dihydropyridine derivatives)	−0.02024 (−0.03450, −0.00571)*
**NM (numeric memory)**			
**ATC level 5 (*R* ^2^=0.1065)**		**ATC level 4 (*R* ^2^=0.1066)**	
N02BE01 (paracetamol)	−0.03647 (−0.06108, −0.00851)*	N02BE (anilides)	−0.03785 (−0.06223, −0.01118)*
N02AA08 (dihydrocodeine)	−0.10960 (−0.20620, −0.00550)*	N02AA (opioids)	−0.07213 (−0.16041, 0.00369)
M01AX05 (glucosamine)	0.04063 (−0.00181, 0.09116)	M01AX (other antiinflammatory and antirheumatic)	0.04016 (−0.00295, 0.09163)
A02BC03 (lansoprazole)	−0.04107 (−0.09278, 0.00197)	A02BC (PPIs)	−0.02203 (−0.05503, 0.00246)
C09AA05 (ramipril)	−0.02513 (−0.07356, 0.00622)	G03AC (progestogens)	0.04972 (−0.00965, 0.13566)
R06AE07 (cetirizine)	0.03371 (−0.01235, 0.10683)	C09AA (ACE inhibitors)	−0.01827 (−0.05453, 0.00485)
B01AA03 (warfarin)	0.03686 (−0.01721, 0.12530)	C01DA (organic nitrates)	−0.04527 (−0.13353, 0.01361)
C03AA01 (bendroflumethiazide)	−0.01574 (−0.05545, 0.00846)	B01AA (vitamin K antagonists)	0.03853 (−0.01769, 0.12602)
R05DA04 (codeine)	−0.02008 (−0.07445, 0.01088)	C10AX (lipid modifying agents)	0.01844 (−0.01003, 0.06785)
C09CA06 (candesartan)	−0.02934 (−0.11006, 0.01766)	R01AD (corticosteroids)	0.02435 (−0.01179, 0.08100)
**PM (prospective memory)**			
**ATC level 5 (*R* ^2^= 0.0943)**		**ATC level 4 (*R* ^2^= 0.0944)**	
N02BE01 (paracetamol)	−0.09979 (−0.13565, −0.06518)* (OR = 0.9050)	N02BE (anilides)	−0.10113 (−0.13560, −0.06558)* (OR = 0.9038)
M01AX05 (glucosamine)	0.10217 (0.01818, 0.17748)* (OR = 1.1076)	M01AX (other antiinflammatory and antirheumatic)	0.11527 (0.04303, 0.18364)* (OR = 1.1222)
M01AE01 (ibuprofen)	0.05692 (0.00747, 0.10080)* (OR = 1.0586)	M01AE (propionic acid derived NSAIDs)	0.06744 (0.02279, 0.10933)* (OR = 1.0698)
N06AA09 (amitriptyline)	−0.10248 (−0.19799, −0.00354)* (OR = 0.9026)	N03AX (other antiepileptics)	−0.14217 (−0.26407, −0.00951)* (OR = 0.8675)
R06AX13 (loratadine)	0.12893 (−0.00544, 0.29157) (OR = 1.1376)	N06AA (nsMRIs)	−0.08146 (−0.16674, −0.00015)* (OR = 0.9218)
R03AC12 (salmeterol)	−0.07555 (−0.21009, 0.01540) (OR = 0.9272)	S01EE (prostaglandin analogues)	0.11824 (−0.00586, 0.27104) (OR = 1.1255)
C03CA01 (furosemide)	−0.07719 (−0.21482, 0.01456) (OR = 0.9257)	M05BA (biphosphonates)	−0.06978 (−0.17472, 0.00927) (OR = 0.9326)
C10AX06 (omega−3)	0.04210 (−0.01127, 0.13193) (OR = 1.0430)	R06AX (systemic antihistamines)	0.07195 (−0.01352, 0.19499) (OR = 1.0746)
C09CA04 (irbesartan)	0.03654 (−0.03393, 0.16861) (OR = 1.0372)	N02CC (triptans)	0.09617 (−0.02375, 0.28778) (OR = 1.1009)
A11CA01 (retinol—vitamin A)	0.01624 (−0.03440, 0.13075) (OR = 1.0164)	C07AA (non‐selective β‐blockers)	0.07365 (−0.01947, 0.22003) (OR = 1.0764)
**PCA‐cognition**			
**ATC level 5 (*R* ^2^= 0.2691)**		**ATC level 4 (*R* ^2^= 0.2695)**	
N02BE01 (paracetamol)	−0.06424 (−0.07664, −0.05190)*	N02BE (anilides)	−0.06422 (−0.07659, −0.05171)*
M01AX05 (glucosamine)	0.05885 (0.03392, 0.08357)*	M01AX (other antiinflammatory and antirheumatic)	0.06603 (0.04270, 0.08935)*
N06AA09 (amitriptyline)	−0.09213 (−0.12632, −0.05705)*	N03AX (other antiepileptics)	−0.12979 (−0.17667, −0.08371)*
M01AE01 (ibuprofen)	0.02949 (0.01531, 0.04360)*	N06AA (nsMRIs)	−0.08311 (−0.11388, −0.05223)*
G03CA57 (conjugated estrogens)	−0.07101 (−0.12168, −0.01575)*	M01AE (propionic acid derived NSAIDs)	0.03360 (0.01967, 0.04740)*
C03CA01 (furosemide)	−0.04771 (−0.09897, 0.00069)	G03CA (estrogens)	−0.04712 (−0.07567, −0.01782)*
M01AE02 (naproxen)	0.04461 (−0.00240, 0.09652)	G03AC (progestogens)	0.07169 (0.02923, 0.11427)*
A10A (insulin)	−0.03938 (−0.09121, 0.00414)	N06AB (SSRIs)	−0.03120 (−0.05871, −0.00385)*
M01AB05 (diclofenac)	0.02367 (−0.00318, 0.05502)	G03DC (estren derivatives)	0.06068 (0.00160, 0.11883)*
A12AA04 (calcium carbonate)	−0.02236 (−0.05616, 0.00406)	C03CA (sulfonamides)	−0.05152 (−0.09994, −0.00225)*

The magnitudes of the effect sizes for the top‐ranked medications ranged between 0.01 and 0.10 Z‐scores, in both positive and negative directions. Only a few medications exhibited effect sizes over 0.10, mostly negative, peaking at >0.20 for valproic acid. Reaction time (RT) was the cognitive variable subject to the largest effect sizes, suggesting that processing speed is especially sensitive to drug effects.

Several analgesics and non‐steroidal anti‐inflammatory products were among the most strongly associated medications across all models. Paracetamol was the top‐ranked medication for all cognitive variables (except RT, where it ranked second), even though its negative effect size was relatively small at around 0.05 Z‐scores (and 10% correct response in PM). Conversely, glucosamine was positively associated with all outcomes by a similar effect size; other anti‐inflammatory products coded under M01AX (specifically chondroitin) were also positively associated. In addition, non‐steroidal anti‐inflammatory drugs (NSAIDs), both propionic acid derivatives (ibuprofen, naproxen) and acetic acid derivatives (diclofenac) were positively associated to most outcomes.

Many medications targeting the CNS were negatively associated with cognitive variables. Amitriptyline was the most consistently ranked, being among the top 10 for all outcomes but numeric memory (NM), with an effect size between 0.03 and 0.08 Z‐scores (for PM: OR = 0.9026). Likewise, the group of antiepileptics coded as N03AX (including topiramate, levetiracetam, lamotrigine, gabapentinoids) yielded a reduction of 0.06 to 0.13 Z‐scores in all outcomes but NM (and OR = 0.8675 for PM); gabapentin specifically worsened RT by 0.06 Z‐scores and valproic acid had the largest effect size for any medication in any cognitive model, with a beta coefficient of 0.20927 (95% credibility interval 0.2682 to 0.14946) in the model for RT.

**FIGURE 1:**
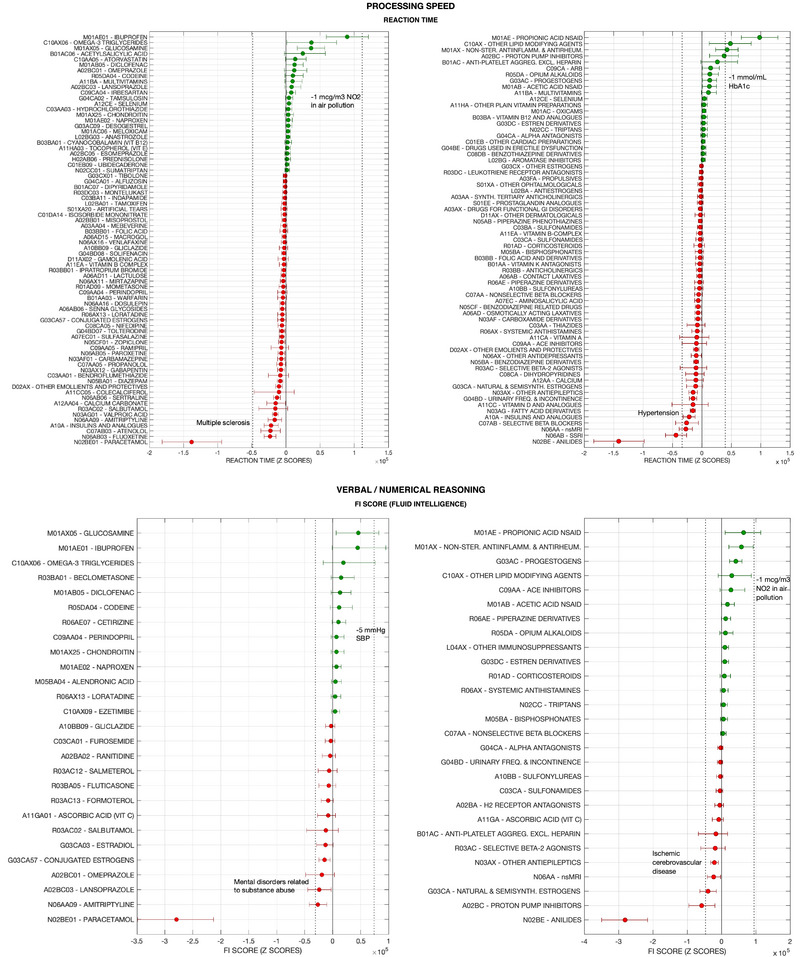
Cognitive footprint of medication according to results from UK Biobank cohort, on the following domains and outcomes: processing speed (reaction time ‐RT‐) and verbal/numerical reasoning (fluid intelligence ‐FI score‐). For each cognitive outcome, the leftward panel presents medications classified according to the last level of the Anatomical Therapeutic Classification (ATC level 5: chemical substances, e.g., ‘ibuprofen’), while the rightward panel groups medications according to the ATC level 4 (pharmacological subgroups, e.g., propionic acid derivatives). Note than while the ATC codes are official, the accompanying terms may have been abbreviated (e.g., ‘tertiary anticholinergics’ for ‘anticholinergics with tertiary amino group’). The cognitive footprint of a medication on a specific cognitive outcome represents the estimated effect of medication use in the UK population (ages 40 to 70), according to the individual effect estimated by modelling UK Biobank data and assuming UK‐wide prevalence of consumption is the same as in the cohort. Units are Z‐scores of the distribution of the cognitive outcome score (RT, FI) across UK Biobank participants. Error bars represent 95% credible intervals. Only medications with over 50% credibility for a non‐zero effect are presented in the graph (i.e., the 50% credible intervals of the corresponding regression coefficient do not contain zero). Negative values and red colour indicate the medication is associated to worse cognitive score, while positive values indicate association to better score. The dashed vertical bars represent the cognitive footprint of other covariates in the model or potential interventions (demographic, medical or environmental conditions, etc.). For instance, the cognitive footprint of multiple sclerosis is calculated as the individual effect of the disease according to the model presented in the same graph (e.g., ATC level 5‐medications and RT), multiplied by the estimated prevalence of the condition in the UK population (ages 40 ‐ 70), extrapolated from the prevalence in the UK Biobank cohort. The cognitive footprint of a hypothetical intervention consisting on reducing air pollution by a certain amount is calculated according to the individual effect of the condition (pollution in this case) according to the model, if it was applied to the entire UK population by the amount indicated (e.g., 1 mcg/m^3^ NO_2_). Abbreviations: Non‐ster.: non‐steroidal; antiinflamm: anti‐inflammatory; antirheum: antirheumatic; ACE: angiotensin‐converting enzyme; NSAID: non‐steroidal anti‐inflammatory drugs; inh.: inhibitors; (semi)synth.: (semi)synthetic; ARB: angiotensin II receptor blockers; SSRI: selective serotonin reuptake inhibitors; freq.: frequency; aggreg.: aggregation; excl.: excluding; nsMRI: non‐selective monoamine reuptake inhibitors.

**FIGURE 2:**
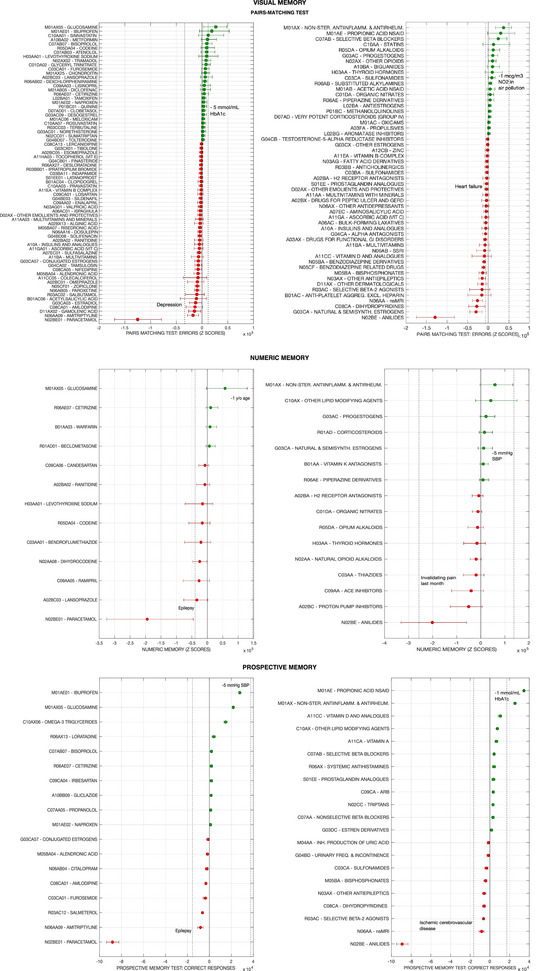
Cognitive footprint of medication according to results from UK Biobank cohort, on the following domains and outcomes: visual memory (pairs‐matching test ‐PaMa‐), numeric memory (NM) and prospective memory). For each cognitive outcome, the leftward panel presents medications classified according to the last level of the Anatomical Therapeutic Classification (ATC level 5: chemical substances, e.g., ‘ibuprofen’), while the rightward panel groups medications according to the ATC level 4 (pharmacological subgroups, e.g., propionic acid derivatives). Note than while the ATC codes are official, the accompanying terms may have been abbreviated (e.g., ‘tertiary anticholinergics’ for ‘anticholinergics with tertiary amino group’). The cognitive footprint of a medication on a specific cognitive outcome represents the estimated effect of medication use in the UK population (ages 40 to 70), according to the individual effect estimated by modelling UK Biobank data and assuming UK‐wide prevalence of consumption is the same as in the cohort. Units are Z‐scores of the distribution of the cognitive outcome score (PaMa, NM, PM) across UK Biobank participants. Error bars represent 95% credible intervals. Only medications with over 50% credibility for a non‐zero effect are presented in the graph (i.e., the 50% credible intervals of the corresponding regression coefficient do not contain zero). Negative values and red colour indicate the medication is associated to worse cognitive score, while positive values indicate association to better score. The dashed vertical bars represent the cognitive footprint of other covariates in the model or potential interventions (demographic, medical or environmental conditions, etc.). For instance, the cognitive footprint of depression is calculated as the individual effect of the disease according to the model presented in the same graph (e.g., ATC level 5‐medications and PaMa), multiplied by the estimated prevalence of the condition in the UK population (ages 40 ‐ 70), extrapolated from the prevalence in the UK Biobank cohort. The cognitive footprint of a hypothetical intervention consisting on reducing Hb1ac levels by a certain amount is calculated according to the individual effect of the condition (Hb1ac blood levels in this case) according to the model, if it was applied to the entire UK population by the amount indicated (e.g., 5 mmol/mL). Abbreviations: Non‐ster.: non‐steroidal; antiinflamm: anti‐inflammatory; antirheum: antirheumatic; ACE: angiotensin‐converting enzyme; NSAID: non‐steroidal anti‐inflammatory drugs; inh.: inhibitors; (semi)synth.: (semi)synthetic; ARB: angiotensin II receptor blockers; SSRI: selective serotonin reuptake inhibitors; freq.: frequency; aggreg.: aggregation; excl.: excluding; nsMRI: non‐selective monoamine reuptake inhibitors.

**FIGURE 3:**
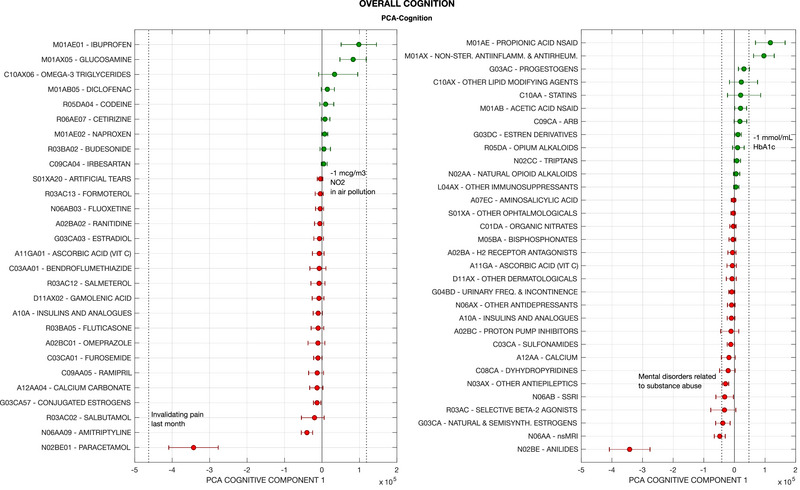
Cognitive footprint of medication according to results from UK Biobank cohort, on ´overall cognition´ (first component of PCA decomposition on the individual test results). The leftward panel presents medications classified according to the last level of the anatomical therapeutic classification (ATC level 5: chemical substances, e.g., ‘ibuprofen’), while the rightward panel groups medications according to the ATC level 4 (pharmacological subgroups, e.g., propionic acid derivatives). Note than while the ATC codes are official, the accompanying terms may have been abbreviated (e.g., ‘tertiary anticholinergics’ for ‘anticholinergics with tertiary amino group’). The cognitive footprint of a medication on a specific cognitive outcome represents the estimated effect of medication use in the UK population (ages 40 to 70), according to the individual effect estimated by modelling UK Biobank data and assuming UK‐wide prevalence of consumption is the same as in the cohort. Units are Z‐scores of the distribution of the cognitive outcome score (PCA‐Cognition) across UK Biobank participants. Error bars represent 95% credible intervals. Only medications with over 50% credibility for a non‐zero effect are presented in the graph (i.e., the 50% credible intervals of the corresponding regression coefficient do not contain zero). Negative values and red colour indicate the medication is associated to worse cognitive score, while positive values indicate association to better score. The dashed vertical bars represent the cognitive footprint of other covariates in the model or potential interventions (demographic, medical or environmental conditions, etc.). For instance, the cognitive footprint of recent invalidating pain is calculated as the individual effect of the condition according to the model presented in the same graph (e.g., ATC level 5‐medications and PCA‐Cognition), multiplied by the estimated prevalence of the condition in the UK population (ages 40 ‐ 70), extrapolated from the prevalence in the UK Biobank cohort. The cognitive footprint of a hypothetical intervention consisting on reducing air pollution by a certain amount is calculated according to the individual effect of the condition (pollution in this case) according to the model, if it was applied to the entire UK population by the amount indicated (e.g., 1 mcg/m^3^ NO_2_). Abbreviations: Non‐ster.: non‐steroidal; antiinflamm: anti‐inflammatory; antirheum: antirheumatic; ACE: angiotensin‐converting enzyme; NSAID: non‐steroidal anti‐inflammatory drugs; inh.: inhibitors; (semi)synth.: (semi)synthetic; ARB: angiotensin II receptor blockers; SSRI: selective serotonin reuptake inhibitors; freq.: frequency; aggreg.: aggregation; excl.: excluding; nsMRI: non‐selective monoamine reuptake inhibitors.

Figures 1 and 2 present the footprint on individual cognitive tests, and Figure 3 on PCA‐Cognition, of all modeled medications for which the 50% credible intervals of the regression coefficients did not enclose zero for reaction time and PCA‐Cognition. Data for all other cognitive variables are presented in Figure S1. The data points represent the quantity of cognitive capital, extrapolated to the UK population aged between 40–70 years, that is potentially lost or gained by regular medication use. This inference rests on two assumptions. First, that the cross‐sectional associations found in the models are directly related to the medication use, and not a product of hidden confounders or mediators. Second, the prevalence of medication use in the overall UK population (between ages 40–70) is the same as in the UK Biobank cohort. Therefore, the numbers provided can only be considered as exploratory upper bounds to the cognitive footprint of a medication.

The cognitive footprint is expressed in terms of Z‐scores, considering the distribution of cognitive test scores in the UK Biobank data; except for PM, where the effect is measured as the predicted change in the number of participants doing the test correctly (at first attempt). Since units are standardized and the cognitive footprint is calculated for the UK population, effect sizes are comparable across models. For reference, the cognitive footprints of other conditions (e.g., multiple sclerosis, depression) or hypothetical interventions (e.g., reduction of air pollution) are presented in the graphs. This is given by the value of the regression coefficient of such covariates (in the same models where the association between medication and cognition is explored).

Of those medications with a positive cognitive footprint, propionic acid NSAIDs (especially ibuprofen) and non‐steroidal anti‐inflammatory medications coded as M01AX (especially glucosamine) have the largest value for most cognitive domains: around 100,000 Z‐scores of PCA‐Cognition in both cases, similar to a general reduction of the population's age by 2 months or 1 mcg/m^3^ NO_2_ in air pollution. Omega‐3 triglycerides also had a positive footprint on several domains (RT, fluid intelligence (FI), PM, PCA‐Cognition).

### 3.2 EPIC Norfolk

Six medications were consumed by over 10% participants in the EPIC Norfolk cohort: bendroflumethiazide, simvastatin, omega‐3, glucosamine, aspirin, and paracetamol. The results show some similarities to those from UK‐Biobank, although many associations are not significant (Figures S1–S4). Paracetamol has a negative footprint, and M01AX drugs (glucosamine and, to a lesser extent, chondroitin) have (amongst) the largest positive footprint for all cognitive outcomes save the Hopkins verbal learning test (HVLT). Omega‐3 triglycerides has relatively large positive footprint for several (four) cognitive outcomes.

### 3.3 CaPS

In the CaPS cohort, five medications were taken by over 10% participants in at least one wave: atenolol, omega‐3 triglycerides, aspirin, and paracetamol. (Figures S5–S8)

Similar to UK Biobank and EPIC, paracetamol (anilides) had among the most negative cognitive footprint on several outcomes: choice RT, Alice Heim group ability test (AH4) and measures of global cognition (CAMCOG and MMSE), ranging between 60,000 and 80,000 Z‐scores (Figure S9).

Omega‐3 triglycerides had a positive footprint. Unlike UK Biobank and EPIC, M01AX drugs had no relevant positive footprint; however, they were far less used in the older CaPS cohort (e.g., glucosamine was not used by anybody in waves 3 and 4, and only by 2.51% in wave 5).

## 4 Discussion

The aim of developing a cognitive footprint metric is to assess not only the effect size (both positive and negative) of an intervention, event or exposure, but also the cumulative impact defined by the duration of that effect and its coverage of the population. The cognitive footprint can be applied across the lifespan to assess both short‐term effects (hours, days, weeks, and months) and effects that may last a lifetime or significantly increase the risk of late life dementia. The concept has been applied in the cognitive impact of neuropsychiatric disease (Cullen et al. 2017).

Medication use provides an opportunity to develop the cognitive footprint concept further. Medications are widely employed, and many have adverse cognitive effects. Cognition is rarely measured during any phase of the trials of interventions in non‐neurological/psychiatric disorders, or in post‐market surveillance. However, both on‐ and off‐target effects on cognition are not confined to neuropsychiatric disease. Ideally, cognitive measures should be employed throughout the R&D process such that where there is parity of outcome on the primary disease effect, the drugs with favorable or least adverse cognitive footprint would guide prescribing advice. In the absence of randomized controlled trial data, we have used the cognitive data and reported medication use from UK Biobank and further validated in the EPIC Norfolk and Caerphilly cohorts.

Many medications have been associated with adverse cognitive effects. In particular those developed for CNS disease, such as anti‐epileptic drugs and anti‐psychotics, or those with anticholinergic effects (Risacher et al. 2016). There are many reports of potential effects on later life dementia risk, for example, in relation to proton pump inhibitors (Gomm et al. 2016; Haenisch et al. 2015). This can be an important additional component of the cognitive footprint of a medication besides short‐term effects (Akter et al. 2015).

Our findings replicate within a Bayesian framework those of Nevado‐Holgado (Nevado‐Holgado et al. 2016), who also interrogated the UK Biobank dataset, and confirm key findings in two additional cohorts. Moreover, we have modeled the potential impact at the national level to provide an upper bound of the effects conditional on the frequencies of prescription and have compared the effect sizes with those reported for neuropsychiatric diseases (Cullen et al. 2017). Paracetamol emerges as a consistent medication with an adverse effect, which, owing to the frequency of use, has a large negative footprint. Previous review of long‐term paracetamol use has also indicated adverse effects (McCrae et al. 2018).

Ibuprofen and glucosamine (and to a lesser extent omega‐3 triglycerides) emerge as medications with consistent beneficial cognitive effects and a recent review suggests that the latter is also associated with reduced all‐cause mortality (King et al. 2020). The underlying mechanisms are not known although the consistent effects of medication on reaction times and measures sensitive to impaired attention suggest a common mechanism. In addition paracetamol and glucosamine have divergent effects on Brain Derived Neurotrophic Factor (BDNF) in rat models with a decrease with long term paracetamol and increase with glucosamine (Lalert et al. 2023; Chou et al. 2020)

These correlative associations cannot prove causation: confounding factors, reverse causation, or selection bias may all have influenced the results across the cohorts; not only is there a risk of reverse causation per se but also of including participants with prodromal neurodegenerative disease (Gallacher et al. 2009; Gallacher et al. 2011). We have, however, used high‐dimensional modeling to account for a wide array of confounders such as the presence of pain. In addition, most of the data are cross‐sectional. Biobank does have serial cognitive tests performed in follow‐up visits, but they are taken by only a small subset of the baseline sample and our exploratory analyses did not yield significant findings. Further, cognitive tests will be available in the future but the limitation will remain that it is difficult to be sure of the total drug exposure during the period of follow up and this may mask effects both for cross sectional and longitudinal analyses. The more robust approach as suggested is to assess cognition routinely in phase 3 randomized trials. The cognitive effects were not seen across all domains. Reaction times and measures sensitive to impaired attention were the most consistently affected, reflecting a common mechanism for drug side‐effects. Further, many drugs will have short‐term effects that will not be captured in our analysis. The importance of cognitive side‐effects argues for consistent assessment during Phase III trials, as recommended by Roiser et al. (2016).

Future research could usefully focus on including standard cognitive tests, particularly those assessing attention, in all interventional drug trials. The significance of the small effect sizes can be evaluated by comparison to the cognitive footprint of factors affecting health such as pollution. The divergent effects of glucosamine and paracetamol could also be explored.

## 5 Conclusion

Cognitive effects of commonly used medications can be substantial at a population level and the negative effects comparable to other modifiable factors such as pollution. Medications with positive cognitive effects are less common, but volume of use is quite high. Given the impact of medications on cognition, particularly in the elderly, it is recommended that all Phase 3 trials—regardless of the primary outcome—routinely assess cognition.

## Author Contributions

**Marta Suárez Pinilla**: Data curation, Formal analysis, Methodology, Validation, Writing–original draft, Writing–review and editing **Charlotte R. Stoner**: Conceptualization, Writing–review and editing **Martin Knapp**: Conceptualization, Formal analysis, Methodology, Writing–review and editing **Parashkev Nachev**: Data curation, Formal analysis, Methodology, Validation, Writing–original draft, Writing–review and editing, Conceptualization, Funding acquisition, Project administration, Supervision **Martin Rossor**: Conceptualization, Funding acquisition, Methodology, Project administration, Supervision, Writing–original draft, Writing–review and editing.

## Ethics Statement

Covered by the individual cohorts

◦ EPIC Norfolk ‐ 1HC, 2HC, record linkage: 98CN01

‐ Pilot phase of 3HC (2004–2006): REC approval 04/Q0101/7

‐ 3HC (2006‐2011): REC approval 05/Q0101/191

‐ 4HC: REC approval 12/EE/0346

‐ 5HC: REC approval 16/EE/0259

◦ Caerphilly Prospective Cohort –

Gwent REC (ref JW/ST/01/69): “The pattern and causes of asymptomatic cognitive decline leading to dementia”

## Conflicts of Interest

The authors declare no conflicts of interest.

### Peer Review

The peer review history for this article is available at https://publons.com/publon/10.1002/brb3.70200.

## Supporting information



Supplementary Figure 1. Cognitive footprint of medication according to results from UK Biobank cohort, on the following domains and outcomes: Abstract Nonverbal Reasoning, Verbal Memory, Visual Processing, Strategic Planning, Trail Making Tests 1 & 2 (Tmt1 & 2). For each cognitive outcome, the leftward panel presents medications classified according to the last level of the Anatomical Therapeutic Classification (ATC level 5: chemical substances, e.g., ‘ibuprofen’), while the rightward panel groups medications according to the ATC level 4 (pharmacological subgroups, e.g., propionic acid derivatives). Note than while the ATC codes are official, the accompanying terms may have been abbreviated (e.g., ‘tertiary anticholinergics’ for ‘anticholinergics with tertiary amino group’). The cognitive footprint of a medication on a specific cognitive outcome represents the estimated effect of medication use in the UK population (ages 40 to 70), according to the individual effect estimated by modeling UK Biobank data and assuming UK‐wide prevalence of consumption is the same as in the cohort. Units are Z‐scores of the distribution of the cognitive outcome score (RT, FI) across UK Biobank participants. Error bars represent 95% credible intervals. Only medications with over 50% credibility for a non‐zero effect are presented in the graph (i.e., the 50% credible intervals of the corresponding regression coefficient do not contain zero). Negative values and red color indicate the medication is associated to worse cognitive score, while positive values indicate association to better score. The dashed vertical bars represent the cognitive footprint of other covariates in the model or potential interventions (demographic, medical or environmental conditions, etc.). For instance, the cognitive footprint of multiple sclerosis is calculated as the individual effect of the disease according to the model presented in the same graph (e.g., ATC level 5‐medications and RT), multiplied by the estimated prevalence of the condition in the UK population (ages 40 ‐ 70), extrapolated from the prevalence in the UK Biobank cohort. The cognitive footprint of a hypothetical intervention consisting on reducing air pollution by a certain amount is calculated according to the individual effect of the condition (pollution in this case) according to the model, if it was applied to the entire UK population by the amount indicated (e.g., 1 mcg/m^3^ NO_2_). Abbreviations: Non‐ster.: non‐steroidal; antiinflamm: anti‐inflammatory; antirheum: antirheumatic; ACE: angiotensin‐converting enzyme; NSAID: non‐steroidal anti‐inflammatory drugs; inh.: inhibitors; (semi)synth.: (semi)synthetic; ARB: angiotensin II receptor blockers; SSRI: selective serotonin reuptake inhibitors; freq.: frequency; aggreg.: aggregation; excl.: excluding; nsMRI: non‐selective monoamine reuptake inhibitors.

Supplementary Figure 2. Cognitive footprint of medication according to results from EPIC Norfolk cohort, on the domain of processing speed, assessed through two outcomes: reaction time in the simple and complex visual sensitivity test (simple and complex VST). For each cognitive outcome, the leftward panel presents medications classified according to the last level of the Anatomical Therapeutic Classification (ATC level 5: chemical substances, e.g., ‘ibuprofen’), while the rightward panel groups medications according to the ATC level 4 (pharmacological subgroups, e.g., propionic acid derivatives). Note than while the ATC codes are official, the accompanying terms may have been abbreviated (e.g., ‘tertiary anticholinergics’ for ‘anticholinergics with tertiary amino group’). The cognitive footprint of a medication on a specific cognitive outcome represents the estimated effect of medication use in the UK population (ages 50 to 90), according to the individual effect estimated by modeling EPIC Norfolk data and assuming UK‐wide prevalence of consumption is the same as in the cohort. Units are Z‐scores of the distribution of the cognitive outcome score (simple/complex VST) across EPIC Norfolk participants. Error bars represent 95% credible intervals. Only medications with over 50% credibility for a non‐zero effect are presented in the graph (i.e., the 50% credible intervals of the corresponding regression coefficient do not contain zero). Negative values and red color indicate the medication is associated to worse cognitive score, while positive values indicate association to better score. Abbreviations: Non‐ster.: non‐steroidal; antiinflamm: anti‐inflammatory; antirheum: antirheumatic; ACE: angiotensin‐converting enzyme; NSAID: non‐steroidal anti‐inflammatory drugs; inh.: inhibitors; (semi)synth.: (semi)synthetic; ARB: angiotensin II receptor blockers; SSRI: selective serotonin reuptake inhibitors; freq.: frequency; aggreg.: aggregation; excl.: excluding; nsMRI: non‐selective monoamine reuptake inhibitors.

Supplementary Figure 3. Cognitive footprint of medication according to results from EPIC Norfolk cohort, on the domains of premorbid IQ (National Adult Reading Test ‐NART‐) and global cognitive function (assessed by two outcomes: Extended Mental State Examination ‐EMSE‐ and Mini Mental State Exam ‐MMSE‐). For each cognitive outcome, the leftward panel presents medications classified according to the last level of the Anatomical Therapeutic Classification (ATC level 5: chemical substances, e.g., ‘ibuprofen’), while the rightward panel groups medications according to the ATC level 4 (pharmacological subgroups, e.g., propionic acid derivatives). Note than while the ATC codes are official, the accompanying terms may have been abbreviated (e.g., ‘tertiary anticholinergics’ for ‘anticholinergics with tertiary amino group’). The cognitive footprint of a medication on a specific cognitive outcome represents the estimated effect of medication use in the UK population (ages 50 to 90), according to the individual effect estimated by modeling EPIC Norfolk data and assuming UK‐wide prevalence of consumption is the same as in the cohort. Units are Z‐scores of the distribution of the cognitive outcome score across EPIC Norfolk participants. Error bars represent 95% credible intervals. Only medications with over 50% credibility for a non‐zero effect are presented in the graph (i.e., the 50% credible intervals of the corresponding regression coefficient do not contain zero). Negative values and red color indicate the medication is associated to worse cognitive score, while positive values indicate association to better score. Abbreviations: Non‐ster.: non‐steroidal; antiinflamm: anti‐inflammatory; antirheum: antirheumatic; ACE: angiotensin‐converting enzyme; NSAID: non‐steroidal anti‐inflammatory drugs; inh.: inhibitors; (semi)synth.: (semi)synthetic; ARB: angiotensin II receptor blockers; SSRI: selective serotonin reuptake inhibitors; freq.: frequency; aggreg.: aggregation; excl.: excluding; nsMRI: non‐selective monoamine reuptake inhibitors.

Supplementary Figure 4. Cognitive footprint of medication according to results from EPIC Norfolk cohort, on the domains of verbal episodic memory (Hopkins Verbal Learning Test ‐HVLT‐), non‐verbal episodic memory (CANTAB‐Paired Associate Learning ‐CANTAB‐PAL‐) and prospective memory. For each cognitive outcome, the leftward panel presents medications classified according to the last level of the Anatomical Therapeutic Classification (ATC level 5: chemical substances, e.g., ‘ibuprofen’), while the rightward panel groups medications according to the ATC level 4 (pharmacological subgroups, e.g., propionic acid derivatives). Note than while the ATC codes are official, the accompanying terms may have been abbreviated (e.g., ‘tertiary anticholinergics’ for ‘anticholinergics with tertiary amino group’). The cognitive footprint of a medication on a specific cognitive outcome represents the estimated effect of medication use in the UK population (ages 50 to 90), according to the individual effect estimated by modeling EPIC Norfolk data and assuming UK‐wide prevalence of consumption is the same as in the cohort. Units are Z‐scores of the distribution of the cognitive outcome score across EPIC Norfolk participants. Error bars represent 95% credible intervals. Only medications with over 50% credibility for a non‐zero effect are presented in the graph (i.e., the 50% credible intervals of the corresponding regression coefficient do not contain zero). Negative values and red color indicate the medication is associated to worse cognitive score, while positive values indicate association to better score. Abbreviations: Non‐ster.: non‐steroidal; antiinflamm: anti‐inflammatory; antirheum: antirheumatic; ACE: angiotensin‐converting enzyme; NSAID: non‐steroidal anti‐inflammatory drugs; inh.: inhibitors; (semi)synth.: (semi)synthetic; ARB: angiotensin II receptor blockers; SSRI: selective serotonin reuptake inhibitors; freq.: frequency; aggreg.: aggregation; excl.: excluding; nsMRI: non‐selective monoamine reuptake inhibitors.

Supplementary Figure 5. Cognitive footprint of medication according to results from EPIC Norfolk cohort, on the domain of attention (Letter Cancellation Test). The leftward panel presents medications classified according to the last level of the Anatomical Therapeutic Classification (ATC level 5: chemical substances, e.g., ‘ibuprofen’), while the rightward panel groups medications according to the ATC level 4 (pharmacological subgroups, e.g., propionic acid derivatives). Note than while the ATC codes are official, the accompanying terms may have been abbreviated (e.g., ‘tertiary anticholinergics’ for ‘anticholinergics with tertiary amino group’). The cognitive footprint of a medication on a specific cognitive outcome represents the estimated effect of medication use in the UK population (ages 50 to 90), according to the individual effect estimated by modeling EPIC Norfolk data and assuming UK‐wide prevalence of consumption is the same as in the cohort. Units are Z‐scores of the distribution of the cognitive outcome score across EPIC Norfolk participants. Error bars represent 95% credible intervals. Only medications with over 50% credibility for a non‐zero effect are presented in the graph (i.e., the 50% credible intervals of the corresponding regression coefficient do not contain zero). Negative values and red color indicate the medication is associated to worse cognitive score, while positive values indicate association to better score. Abbreviations: Non‐ster.: non‐steroidal; antiinflamm: anti‐inflammatory; antirheum: antirheumatic; ACE: angiotensin‐converting enzyme; NSAID: non‐steroidal anti‐inflammatory drugs; inh.: inhibitors; (semi)synth.: (semi)synthetic; ARB: angiotensin II receptor blockers; SSRI: selective serotonin reuptake inhibitors; freq.: frequency; aggreg.: aggregation; excl.: excluding; nsMRI: non‐selective monoamine reuptake inhibitors.

Supplementary Figure 6. Cognitive footprint of medication according to results from Caerphilly Prospective Cohort (CaPS), on the domains of processing speed (choice reaction time ‐CRT‐) and verbal/numerical reasoning (Alice Heim Group Ability Test ‐AH4‐). For each cognitive outcome, the leftward panel presents medications classified according to the last level of the Anatomical Therapeutic Classification (ATC level 5: chemical substances, e.g., ‘ibuprofen’), while the rightward panel groups medications according to the ATC level 4 (pharmacological subgroups, e.g., propionic acid derivatives). Note than while the ATC codes are official, the accompanying terms may have been abbreviated (e.g., ‘tertiary anticholinergics’ for ‘anticholinergics with tertiary amino group’). The cognitive footprint of a medication on a specific cognitive outcome represents the estimated effect of medication use in the UK population (ages 55 to 80), according to the individual effect estimated by modeling CaPS data and assuming UK‐wide prevalence of consumption is the same as in the cohort. Units are Z‐scores of the distribution of the cognitive outcome score (CRT, AH4) across CaPS participants. Error bars represent 95% credible intervals. Only medications with over 50% credibility for a non‐zero effect are presented in the graph (i.e., the 50% credible intervals of the corresponding regression coefficient do not contain zero). Negative values and red color indicate the medication is associated to worse cognitive score, while positive values indicate association to better score. Abbreviations: Non‐ster.: non‐steroidal; antiinflamm: anti‐inflammatory; antirheum: antirheumatic; ACE: angiotensin‐converting enzyme; NSAID: non‐steroidal anti‐inflammatory drugs; inh.: inhibitors; (semi)synth.: (semi)synthetic; ARB: angiotensin II receptor blockers; SSRI: selective serotonin reuptake inhibitors; freq.: frequency; aggreg.: aggregation; excl.: excluding; nsMRI: non‐selective monoamine reuptake inhibitors.

Supplementary Figure 7. Cognitive footprint of medication according to results from Caerphilly Prospective Cohort (CaPS), on the domains of premorbid IQ (National Adult Reading Test ‐NART‐) and global cognitive function (assessed through two outcomes: Cambridge Cognitive Examination ‐CAMCOG‐ and Mini Mental State Examination ‐MMSE‐). For each cognitive outcome, the leftward panel presents medications classified according to the last level of the Anatomical Therapeutic Classification (ATC level 5: chemical substances, e.g., ‘ibuprofen’), while the rightward panel groups medications according to the ATC level 4 (pharmacological subgroups, e.g., propionic acid derivatives). Note than while the ATC codes are official, the accompanying terms may have been abbreviated (e.g., ‘tertiary anticholinergics’ for ‘anticholinergics with tertiary amino group’). The cognitive footprint of a medication on a specific cognitive outcome represents the estimated effect of medication use in the UK population (ages 55 to 80), according to the individual effect estimated by modeling CaPS data and assuming UK‐wide prevalence of consumption is the same as in the cohort. Units are Z‐scores of the distribution of the cognitive outcome score (NART, CAMCOG, MMSE) across CaPS participants. Error bars represent 95% credible intervals. Only medications with over 50% credibility for a non‐zero effect are presented in the graph (i.e., the 50% credible intervals of the corresponding regression coefficient do not contain zero). Negative values and red color indicate the medication is associated to worse cognitive score, while positive values indicate association to better score. Abbreviations: Non‐ster.: non‐steroidal; antiinflamm: anti‐inflammatory; antirheum: antirheumatic; ACE: angiotensin‐converting enzyme; NSAID: non‐steroidal anti‐inflammatory drugs; inh.: inhibitors; (semi)synth.: (semi)synthetic; ARB: angiotensin II receptor blockers; SSRI: selective serotonin reuptake inhibitors; freq.: frequency; aggreg.: aggregation; excl.: excluding; nsMRI: non‐selective monoamine reuptake inhibitors.

Supplementary Figure 8. Cognitive footprint of medication according to results from Caerphilly Prospective Cohort (CaPS), on the domains of immediate recall (Rivermead Behavioural Memory Test for immediate recall ‐IMM‐R‐), delayed recall (Rivermead Behavioural Memory Test for delayed recall ‐DEL‐R‐) and prompted recall (Rivermead Behavioural Memory Test for prompted recall ‐PROMPT‐R‐). For each cognitive outcome, the leftward panel presents medications classified according to the last level of the Anatomical Therapeutic Classification (ATC level 5: chemical substances, e.g., ‘ibuprofen’), while the rightward panel groups medications according to the ATC level 4 (pharmacological subgroups, e.g., propionic acid derivatives). Note than while the ATC codes are official, the accompanying terms may have been abbreviated (e.g., ‘tertiary anticholinergics’ for ‘anticholinergics with tertiary amino group’). The cognitive footprint of a medication on a specific cognitive outcome represents the estimated effect of medication use in the UK population (ages 55 to 80), according to the individual effect estimated by modeling CaPS data and assuming UK‐wide prevalence of consumption is the same as in the cohort. Units are Z‐scores of the distribution of the cognitive outcome score (IMM‐R, DEL‐R, PROMPT‐R) across CaPS participants. Error bars represent 95% credible intervals. Only medications with over 50% credibility for a non‐zero effect are presented in the graph (i.e., the 50% credible intervals of the corresponding regression coefficient do not contain zero). Negative values and red color indicate the medication is associated to worse cognitive score, while positive values indicate association to better score. Abbreviations: Non‐ster.: non‐steroidal; antiinflamm: anti‐inflammatory; antirheum: antirheumatic; ACE: angiotensin‐converting enzyme; NSAID: non‐steroidal anti‐inflammatory drugs; inh.: inhibitors; (semi)synth.: (semi)synthetic; ARB: angiotensin II receptor blockers; SSRI: selective serotonin reuptake inhibitors; freq.: frequency; aggreg.: aggregation; excl.: excluding; nsMRI: non‐selective monoamine reuptake inhibitors.

Supplementary Figure 9. Cognitive footprint of medication according to results from Caerphilly Prospective Cohort (CaPS), on the domains of incidental memory and prospective memory. For each cognitive outcome, the leftward panel presents medications classified according to the last level of the Anatomical Therapeutic Classification (ATC level 5: chemical substances, e.g., ‘ibuprofen’), while the rightward panel groups medications according to the ATC level 4 (pharmacological subgroups, e.g., propionic acid derivatives). Note than while the ATC codes are official, the accompanying terms may have been abbreviated (e.g., ‘tertiary anticholinergics’ for ‘anticholinergics with tertiary amino group’). The cognitive footprint of a medication on a specific cognitive outcome represents the estimated effect of medication use in the UK population (ages 55 to 80), according to the individual effect estimated by modeling CaPS data and assuming UK‐wide prevalence of consumption is the same as in the cohort. Units are Z‐scores of the distribution of the cognitive outcome score across CaPS participants. Error bars represent 95% credible intervals. Only medications with over 50% credibility for a non‐zero effect are presented in the graph (i.e., the 50% credible intervals of the corresponding regression coefficient do not contain zero). Negative values and red color indicate the medication is associated to worse cognitive score, while positive values indicate association to better score. Abbreviations: Non‐ster.: non‐steroidal; antiinflamm: anti‐inflammatory; antirheum: antirheumatic; ACE: angiotensin‐converting enzyme; NSAID: non‐steroidal anti‐inflammatory drugs; inh.: inhibitors; (semi)synth.: (semi)synthetic; ARB: angiotensin II receptor blockers; SSRI: selective serotonin reuptake inhibitors; freq.: frequency; aggreg.: aggregation; excl.: excluding; nsMRI: non‐selective monoamine reuptake inhibitors.

Supporting Information

Supporting Information

## Data Availability

Raw data from the three cohorts studied are available via the Dementia Platform UK
