# Supplementary material for: The Cognitive Footprint of Medication Use
Source: Brain Behav. 2025 Jan 19;15(1):e70200. doi: 10.1002/brb3.70200 (PMC11743989; doi:10.1002/brb3.70200)
Supplement: Supplementary file 1 — Supplementary Figure 1. Cognitive footprint of medication according to results from UK Biobank cohort, on the following domains and outcomes: Abstract Nonverbal Reasoning, Verbal Memory, Visual Processing, Strategic Planning, Trail Making Tests 1 & 2 (Tmt1 & 2). For each cognitive outcome, the leftward panel presents medications classified according to the last level of the Anatomical Therapeutic Classification (ATC level 5: chemical substances, e.g., ‘ibuprofen’), while the rightward panel groups medications according to the ATC level 4 (pharmacological subgroups, e.g., propionic acid derivatives). Note than while the ATC codes are official, the accompanying terms may have been abbreviated (e.g., ‘tertiary anticholinergics’ for ‘anticholinergics with tertiary amino group’). The cognitive footprint of a medication on a specific cognitive outcome represents the estimated effect of medication use in the UK population (ages 40 to 70), according to the individual effect estimated by modeling UK Biobank data and assuming UK‐wide prevalence of consumption is the same as in the cohort. Units are Z‐scores of the distribution of the cognitive outcome score (RT, FI) across UK Biobank participants. Error bars represent 95% credible intervals. Only medications with over 50% credibility for a non‐zero effect are presented in the graph (i.e., the 50% credible intervals of the corresponding regression coefficient do not contain zero). Negative values and red color indicate the medication is associated to worse cognitive score, while positive values indicate association to better score. The dashed vertical bars represent the cognitive footprint of other covariates in the model or potential interventions (demographic, medical or environmental conditions, etc.). For instance, the cognitive footprint of multiple sclerosis is calculated as the individual effect of the disease according to the model presented in the same graph (e.g., ATC level 5‐medications and RT), multiplied by the es [file BRB3-15-e70200-s008.pdf]

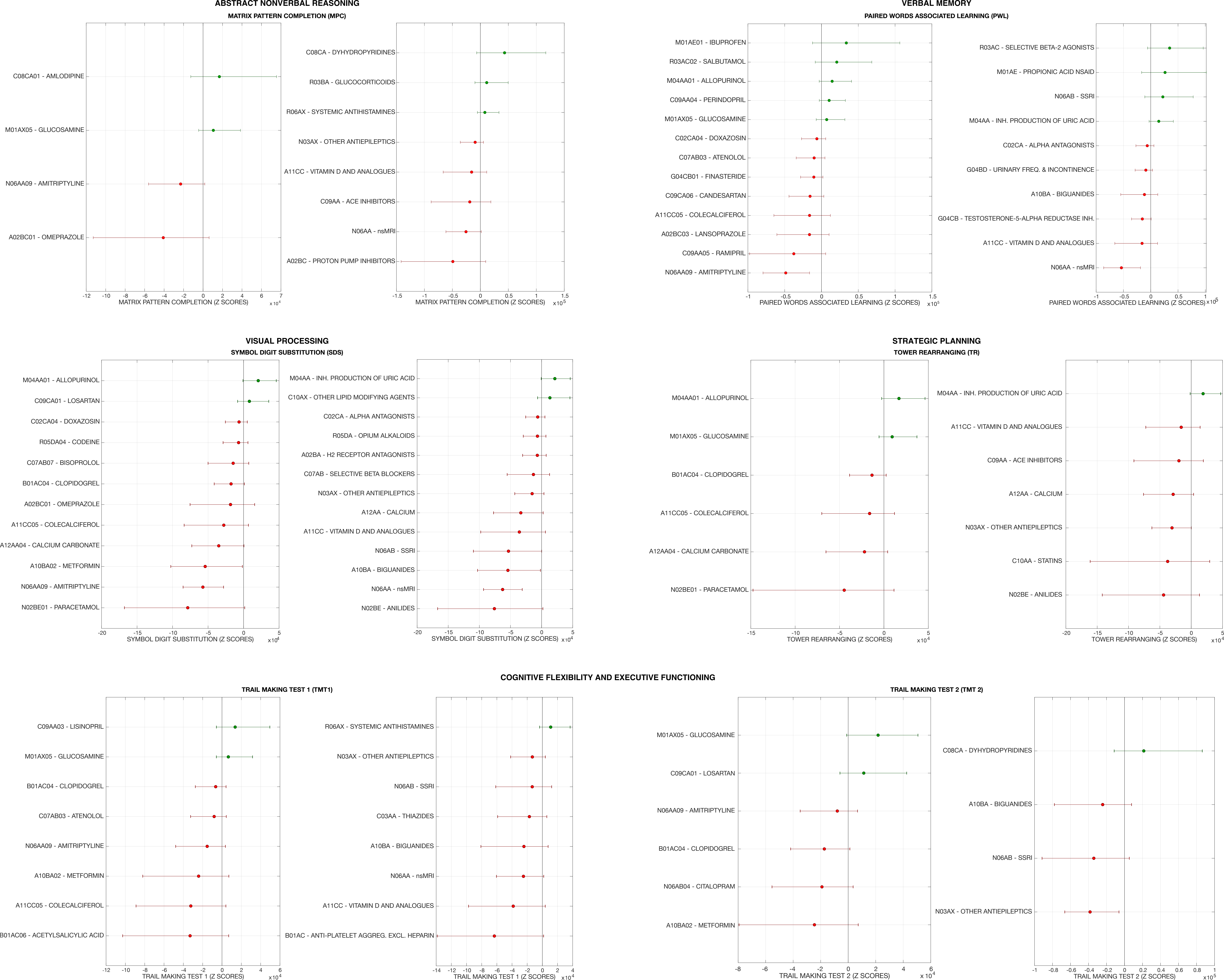

ABSTRACT NONVERBAL REASONING
MATRIX PATTERN COMPLETION (MPC)
C08CA01 - AMLODIPINE
M01AX05 - GLUCOSAMINE
N06AA09 - AMITRIPTYLINE
A02BC01 - OMEPRAZOLE
MATRIX PATTERN COMPLETION (Z SCORES)
C08CA - DYHYDROPYRIDINES
R03BA - GLUCOCORTICOIDS
R06AX - SYSTEMIC ANTIHISTAMINES
N03AX - OTHER ANTIEPILEPTICS
A11CC - VITAMIN D AND ANALOGUES
C09AA - ACE INHIBITORS
N06AA - nsMRI
A02BC - PROTON PUMP INHIBITORS
MATRIX PATTERN COMPLETION (Z SCORES)
VERBAL MEMORY
PAIRED WORDS ASSOCIATED LEARNING (PWL)
M01AE01 - IBUPROFEN
R03AC02 - SALBUTAMOL
M04AA01 - ALLOPURINOL
C09AA04 - PERINDOPRIL
M01AX05 - GLUCOSAMINE
C02CA04 - DOXAZOSIN
C07AB03 - ATENOLOL
G04CB01 - FINASTERIDE
C09CA06 - CANDESARTAN
A11CC05 - COLECALCIFEROL
A02BC03 - LANSOPRAZOLE
C09AA05 - RAMIPRIL
N06AA09 - AMITRIPTYLINE
PAIRED WORDS ASSOCIATED LEARNING (Z SCORES)
R03AC - SELECTIVE BETA-2 AGONISTS
M01AE - PROPIONIC ACID NSAID
N06AB - SSRI
M04AA - INH. PRODUCTION OF URIC ACID
C02CA - ALPHA ANTAGONISTS
G04BD - URINARY FREQ. & INCONTINENCE
A10BA - BIGUANIDES
G04CB - TESTOSTERONE-5-ALPHA REDUCTASE INH.
A11CC - VITAMIN D AND ANALOGUES
N06AA - nsMRI
PAIRED WORDS ASSOCIATED LEARNING (Z SCORES)
VISUAL PROCESSING
SYMBOL DIGIT SUBSTITUTION (SDS)
M04AA01 - ALLOPURINOL
C09CA01 - LOSARTAN
C02CA04 - DOXAZOSIN
R05DA04 - CODEINE
C07AB07 - BISOPROLOL
B01AC04 - CLOPIDOGREL
A02BC01 - OMEPRAZOLE
A11CC05 - COLECALCIFEROL
A12AA04 - CALCIUM CARBONATE
A10BA02 - METFORMIN
N06AA09 - AMITRIPTYLINE
N02BE01 - PARACETAMOL
SYMBOL DIGIT SUBSTITUTION (Z SCORES)
M04AA - INH. PRODUCTION OF URIC ACID
C10AX - OTHER LIPID MODIFYING AGENTS
C02CA - ALPHA ANTAGONISTS
R05DA - OPIUM ALKALOIDS
A02BA - H2 RECEPTOR ANTAGONISTS
C07AB - SELECTIVE BETA BLOCKERS
N03AX - OTHER ANTIEPILEPTICS
A12AA - CALCIUM
A11CC - VITAMIN D AND ANALOGUES
N06AB - SSRI
A10BA - BIGUANIDES
N06AA - nsMRI
N02BE - ANILIDES
SYMBOL DIGIT SUBSTITUTION (Z SCORES)
STRATEGIC PLANNING
TOWER REARRANGING (TR)
M04AA01 - ALLOPURINOL
M01AX05 - GLUCOSAMINE
B01AC04 - CLOPIDOGREL
A11CC05 - COLECALCIFEROL
A12AA04 - CALCIUM CARBONATE
N02BE01 - PARACETAMOL
TOWER REARRANGING (Z SCORES)
M04AA - INH. PRODUCTION OF URIC ACID
A11CC - VITAMIN D AND ANALOGUES
C09AA - ACE INHIBITORS
A12AA - CALCIUM
N03AX - OTHER ANTIEPILEPTICS
C10AA - STATINS
N02BE - ANILIDES
TOWER REARRANGING (Z SCORES)
COGNITIVE FLEXIBILITY AND EXECUTIVE FUNCTIONING
TRAIL MAKING TEST 1 (TMT1)
C09AA03 - LISINOPRIL
M01AX05 - GLUCOSAMINE
B01AC04 - CLOPIDOGREL
C07AB03 - ATENOLOL
N06AA09 - AMITRIPTYLINE
A10BA02 - METFORMIN
A11CC05 - COLECALCIFEROL
B01AC06 - ACETYLSALICYLIC ACID
TRAIL MAKING TEST 1 (Z SCORES)
R06AX - SYSTEMIC ANTIHISTAMINES
N03AX - OTHER ANTIEPILEPTICS
N06AB - SSRI
C03AA - THIAZIDES
A10BA - BIGUANIDES
N06AA - nsMRI
A11CC - VITAMIN D AND ANALOGUES
B01AC - ANTI-PLATELET AGGREG. EXCL. HEPARIN
TRAIL MAKING TEST 1 (Z SCORES)
TRAIL MAKING TEST 2 (TMT 2)
M01AX05 - GLUCOSAMINE
C09CA01 - LOSARTAN
N06AA09 - AMITRIPTYLINE
B01AC04 - CLOPIDOGREL
N06AB04 - CITALOPRAM
A10BA02 - METFORMIN
TRAIL MAKING TEST 2 (Z SCORES)
C08CA - DYHYDROPYRIDINES
A10BA - BIGUANIDES
N06AB - SSRI
N03AX - OTHER ANTIEPILEPTICS
TRAIL MAKING TEST 2 (Z SCORES)
