# Supplementary material for: The Cognitive Footprint of Medication Use
Source: Brain Behav. 2025 Jan 19;15(1):e70200. doi: 10.1002/brb3.70200 (PMC11743989; doi:10.1002/brb3.70200)

VERBAL EPISODIC MEMORY

HOPKINS VERBAL LEARNING TEST

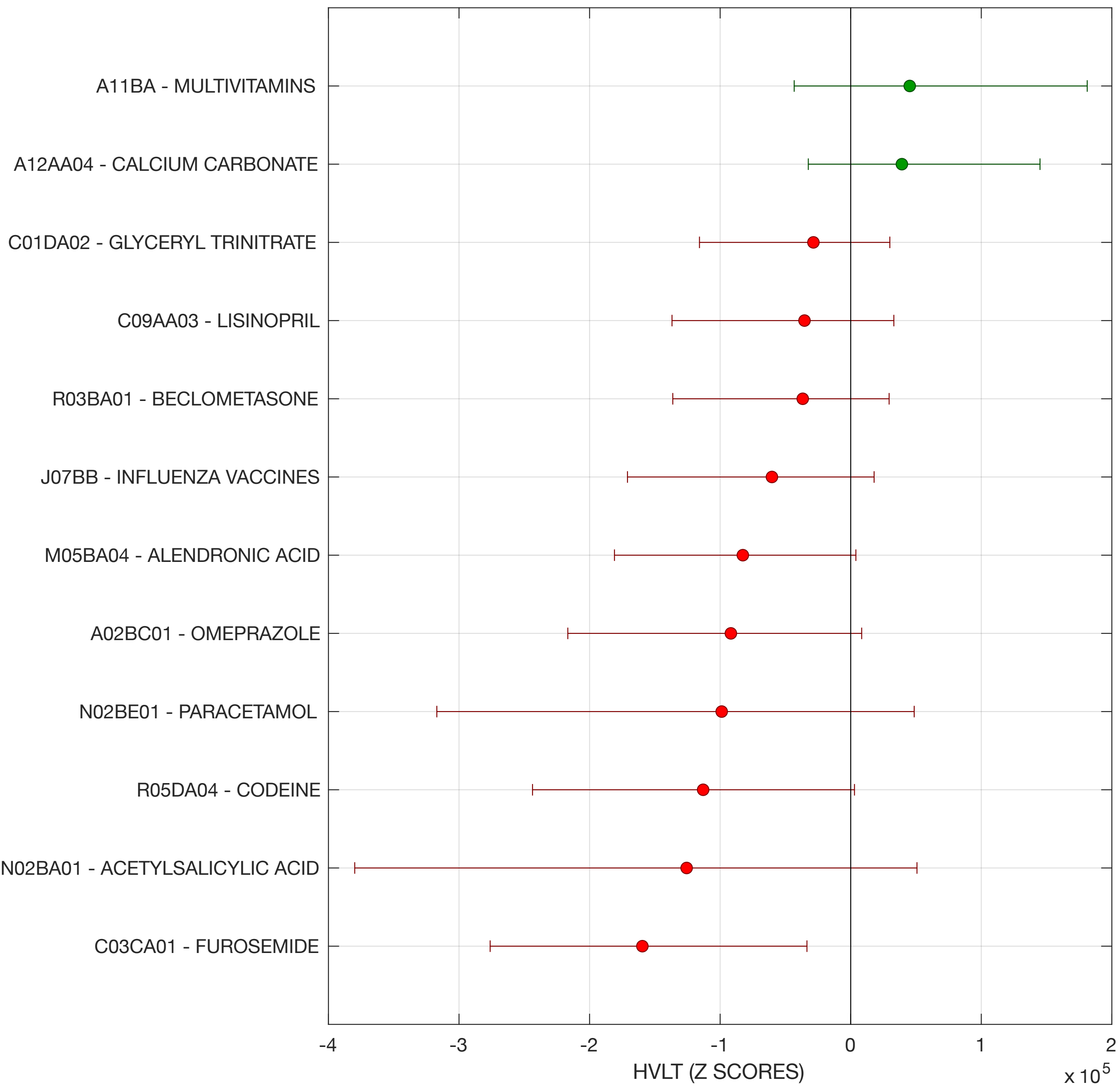

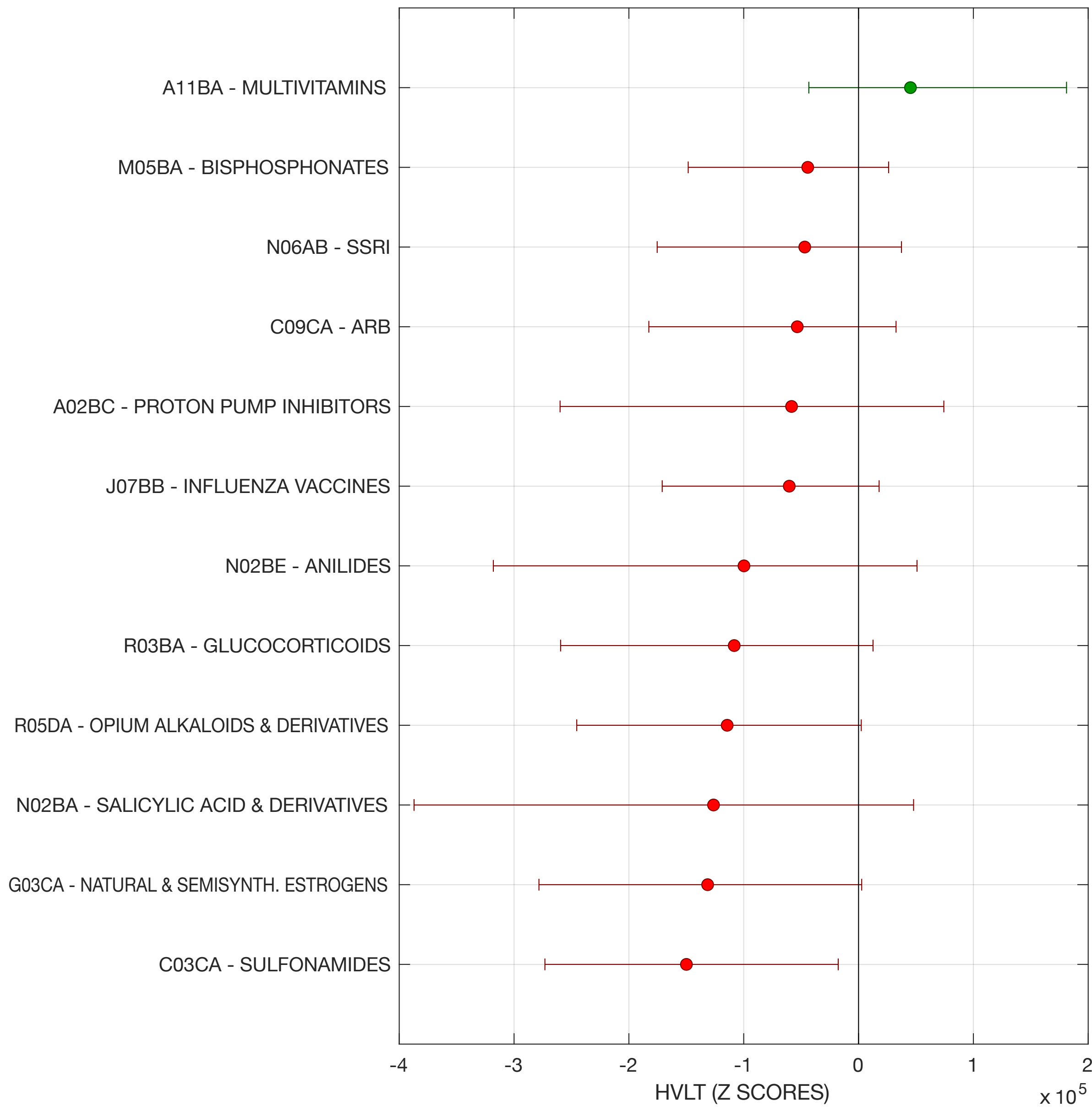

NON-VERBAL EPISODIC MEMORY

CANTAB - PAIRED ASSOCIATED LEARNING (CANTAB-PAL)

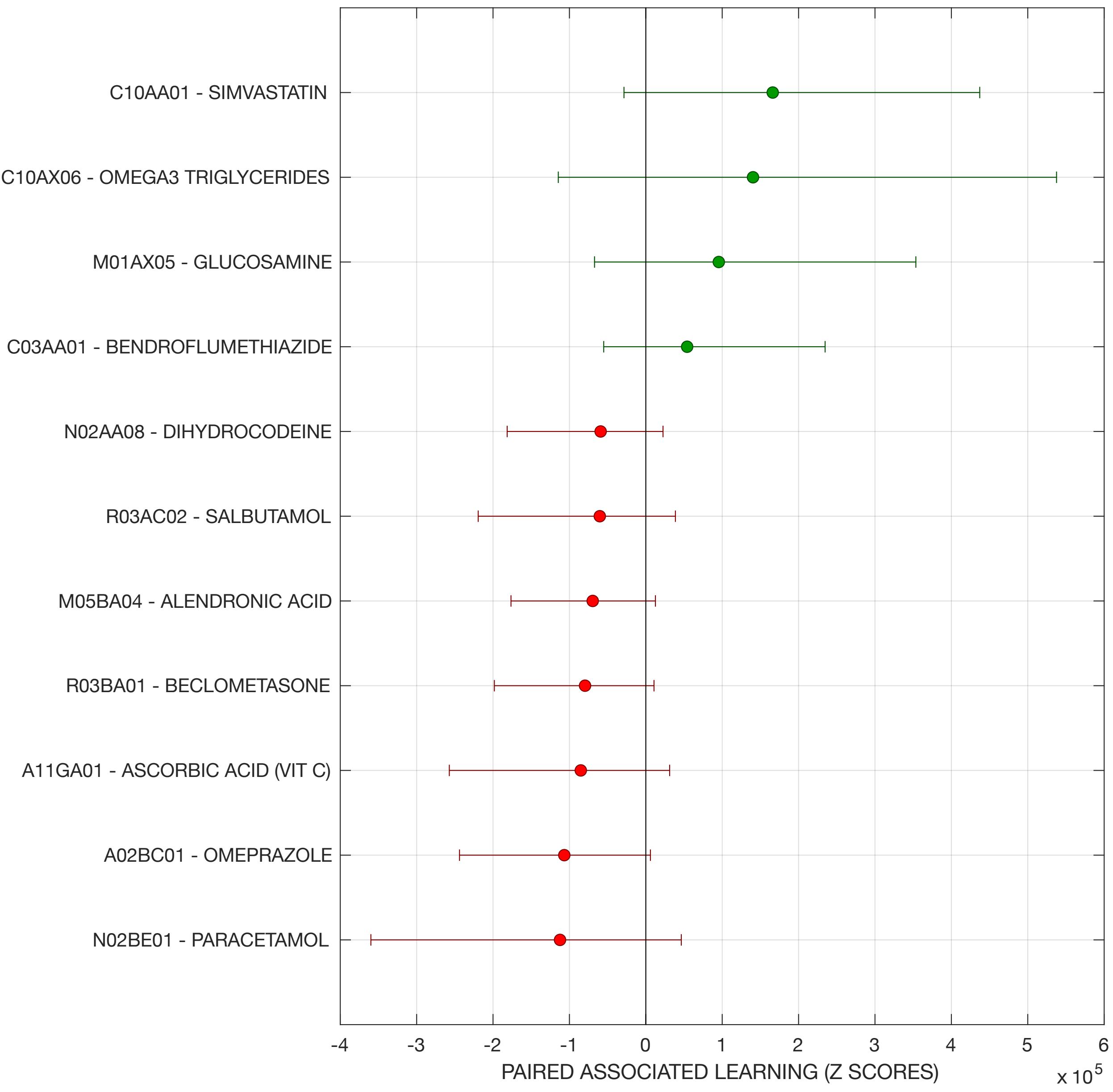

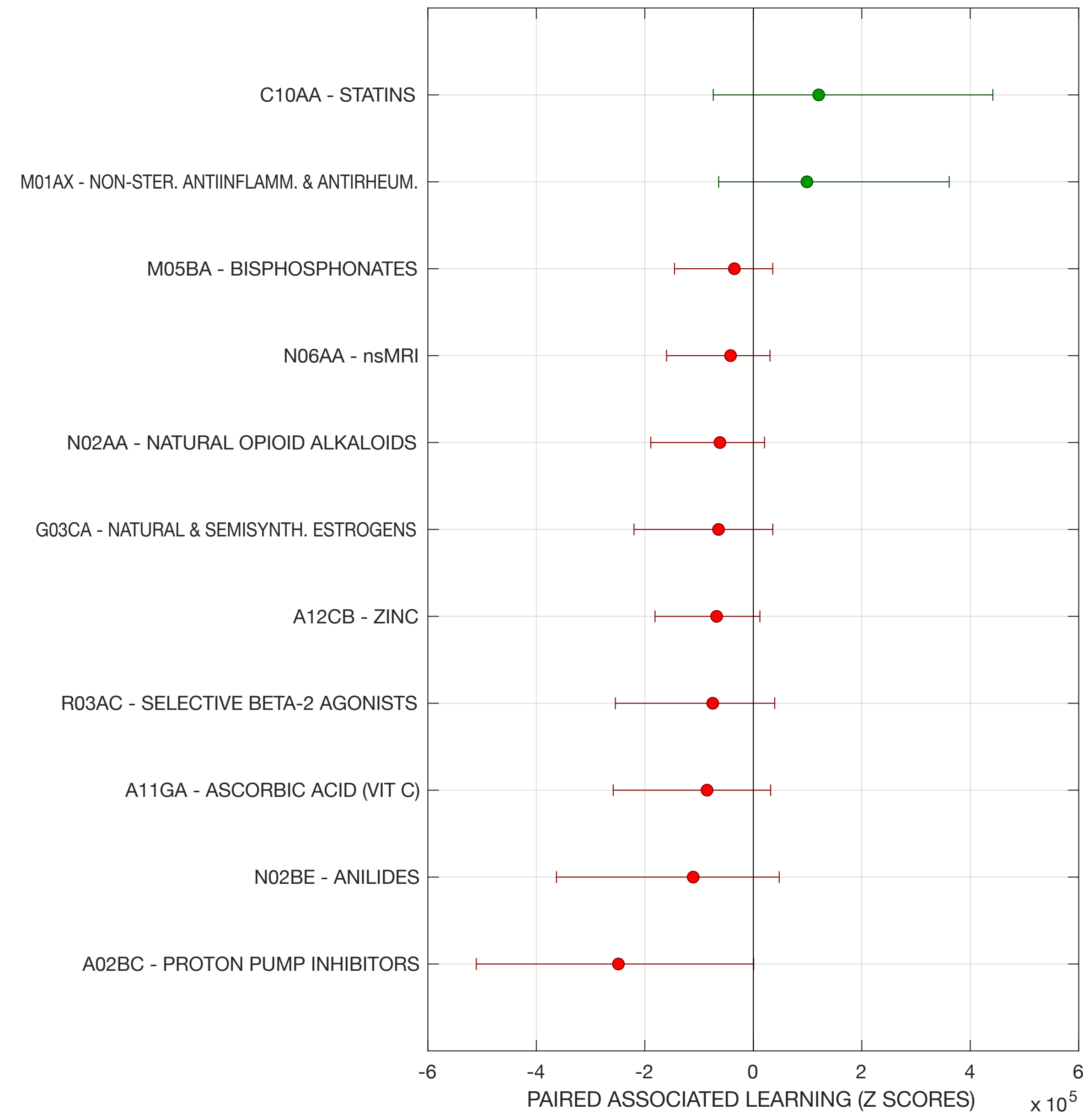

PROSPECTIVE MEMORY

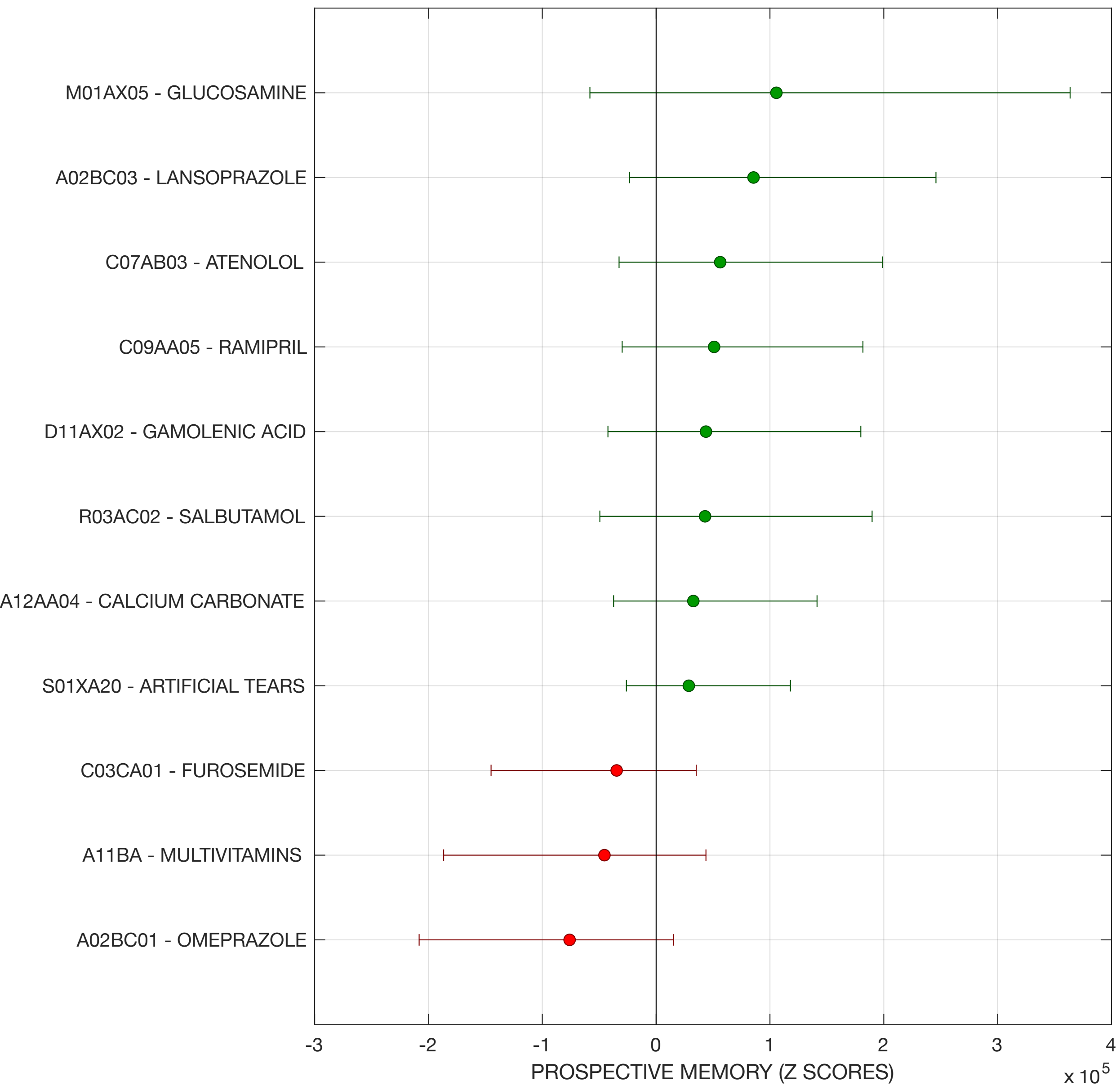

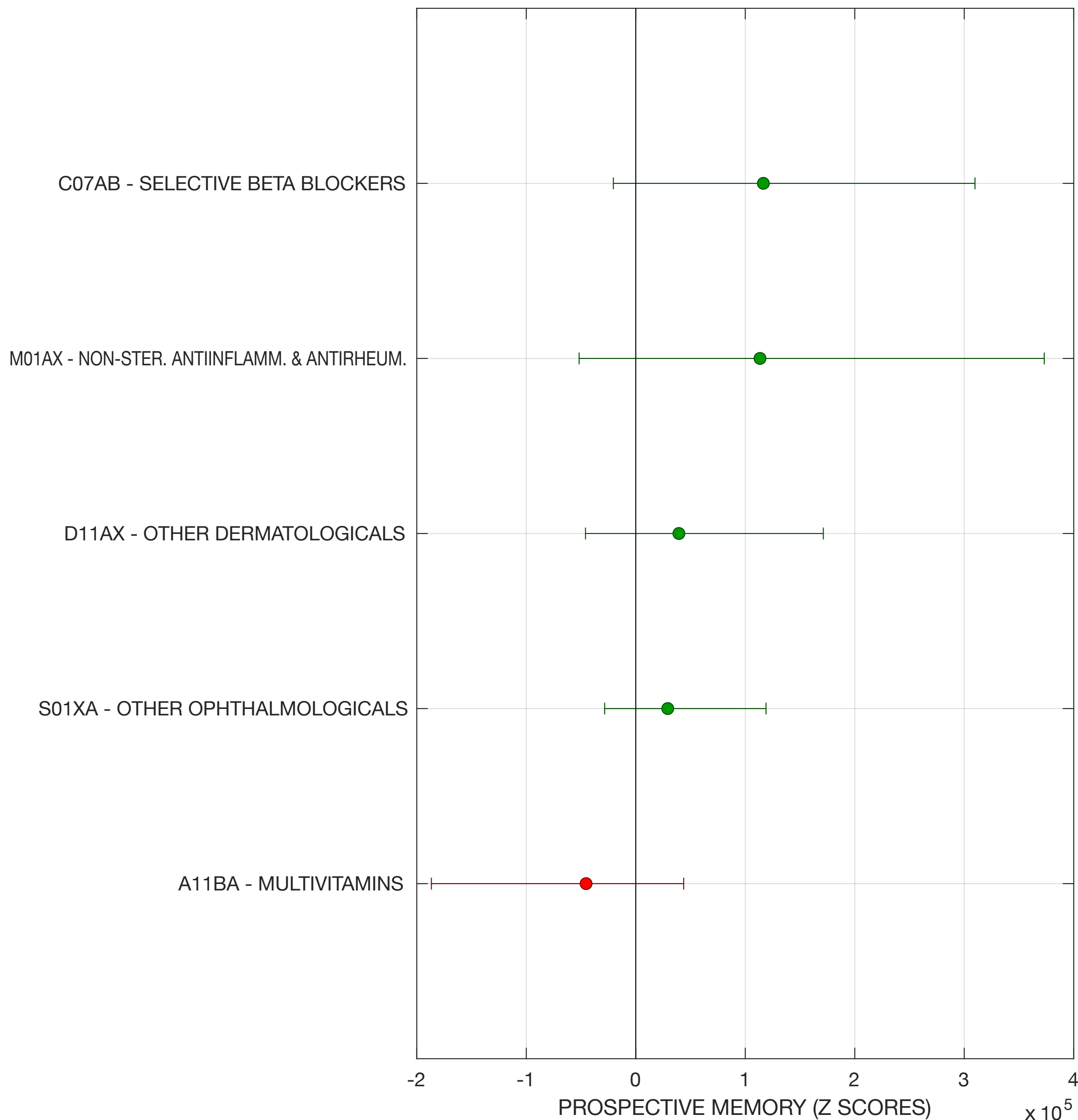

Supplement: Supplementary file 4 — Supplementary Figure 4. Cognitive footprint of medication according to results from EPIC Norfolk cohort, on the domains of verbal episodic memory (Hopkins Verbal Learning Test ‐HVLT‐), non‐verbal episodic memory (CANTAB‐Paired Associate Learning ‐CANTAB‐PAL‐) and prospective memory. For each cognitive outcome, the leftward panel presents medications classified according to the last level of the Anatomical Therapeutic Classification (ATC level 5: chemical substances, e.g., ‘ibuprofen’), while the rightward panel groups medications according to the ATC level 4 (pharmacological subgroups, e.g., propionic acid derivatives). Note than while the ATC codes are official, the accompanying terms may have been abbreviated (e.g., ‘tertiary anticholinergics’ for ‘anticholinergics with tertiary amino group’). The cognitive footprint of a medication on a specific cognitive outcome represents the estimated effect of medication use in the UK population (ages 50 to 90), according to the individual effect estimated by modeling EPIC Norfolk data and assuming UK‐wide prevalence of consumption is the same as in the cohort. Units are Z‐scores of the distribution of the cognitive outcome score across EPIC Norfolk participants. Error bars represent 95% credible intervals. Only medications with over 50% credibility for a non‐zero effect are presented in the graph (i.e., the 50% credible intervals of the corresponding regression coefficient do not contain zero). Negative values and red color indicate the medication is associated to worse cognitive score, while positive values indicate association to better score. Abbreviations: Non‐ster.: non‐steroidal; antiinflamm: anti‐inflammatory; antirheum: antirheumatic; ACE: angiotensin‐converting enzyme; NSAID: non‐steroidal anti‐inflammatory drugs; inh.: inhibitors; (semi)synth.: (semi)synthetic; ARB: angiotensin II receptor blockers; SSRI: selective serotonin reuptake inhibitors; freq.: frequency; aggreg.: aggregation; excl.: excluding; nsMRI: non‐s [file BRB3-15-e70200-s011.pdf]
