# Supplementary material for: The Cognitive Footprint of Medication Use
Source: Brain Behav. 2025 Jan 19;15(1):e70200. doi: 10.1002/brb3.70200 (PMC11743989; doi:10.1002/brb3.70200)

PROCESSING SPEED

CHOICE REACTION TIME

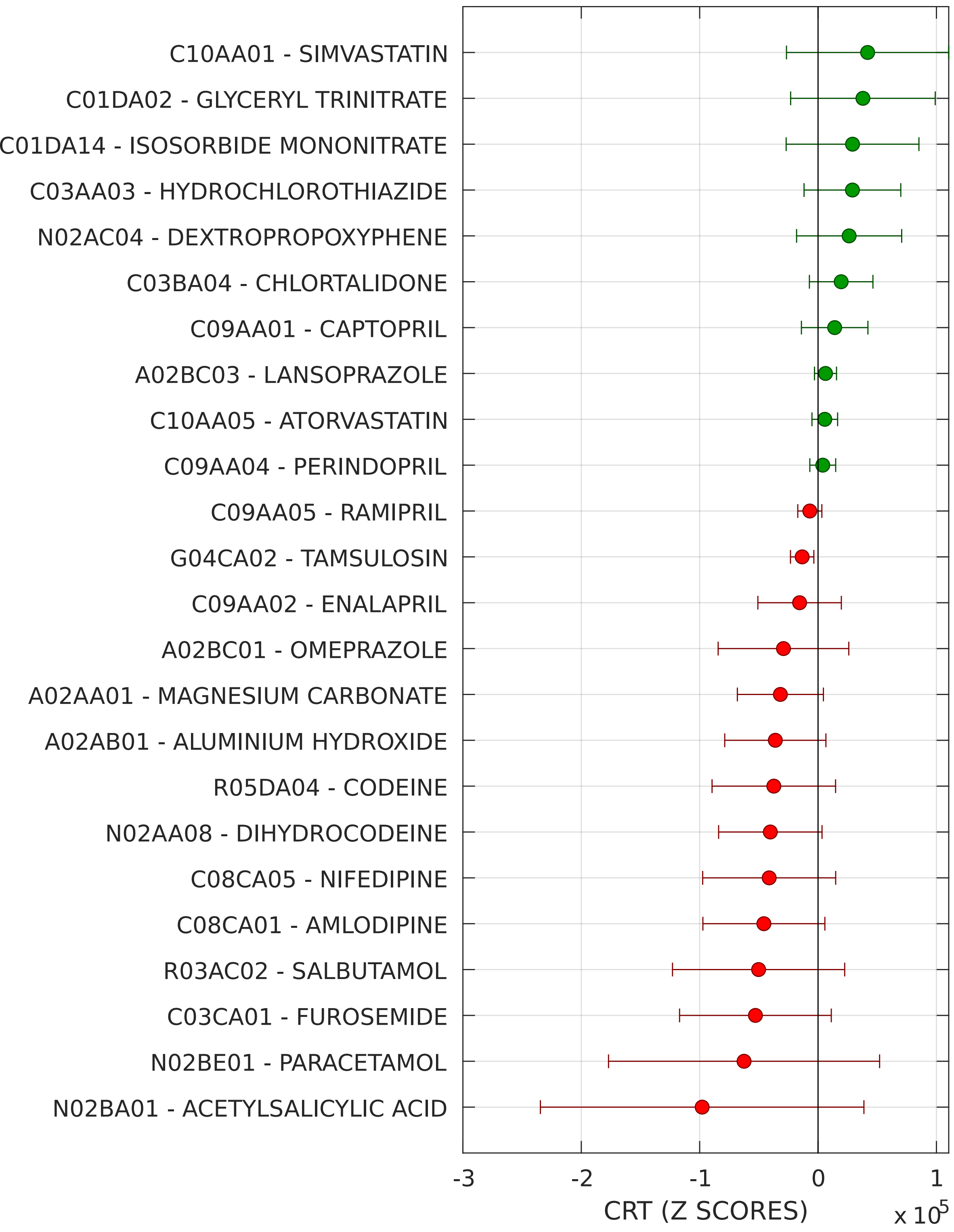

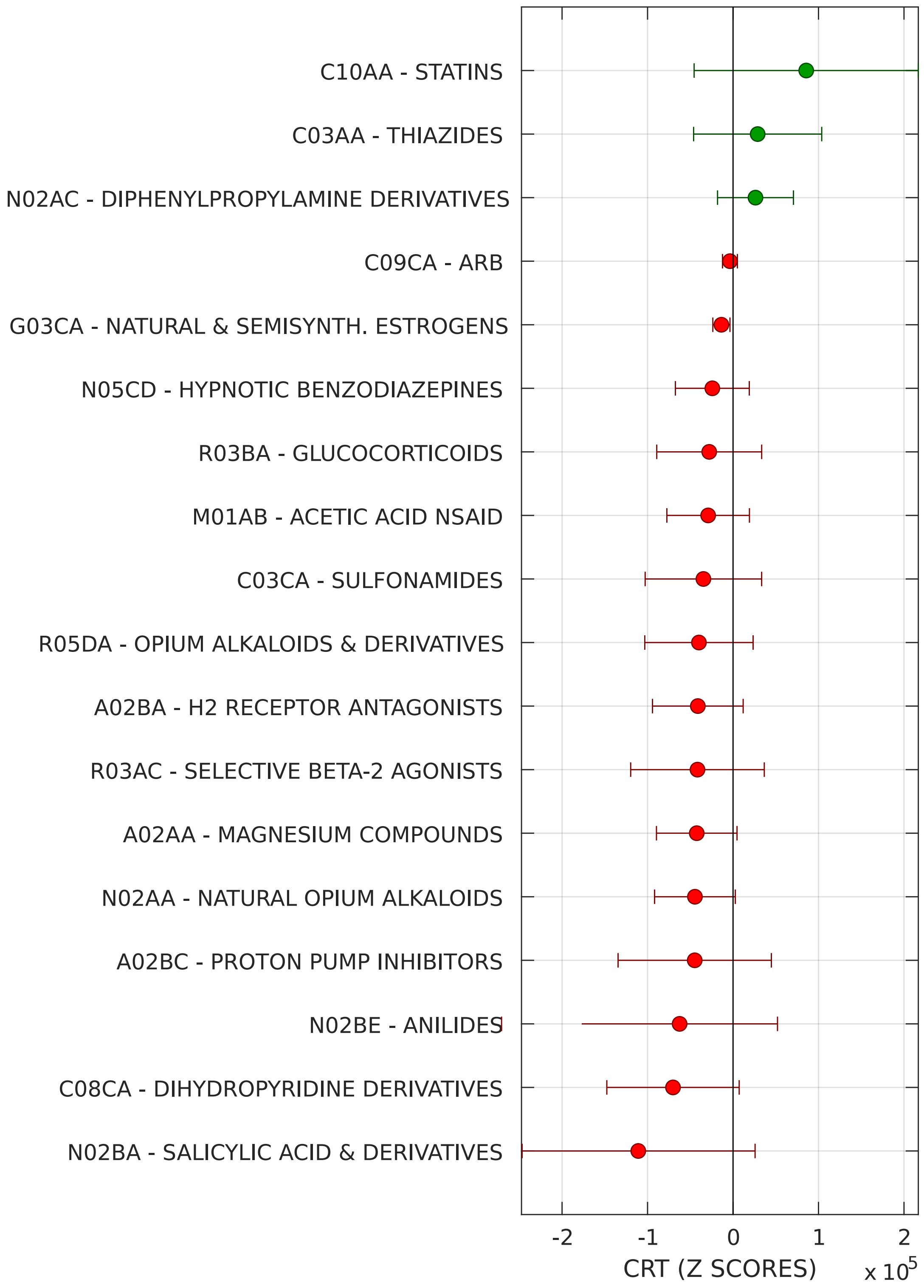

VERBAL / NUMERICAL REASONING

AH4 (ALICE HEIM GROUP ABILITY TEST)

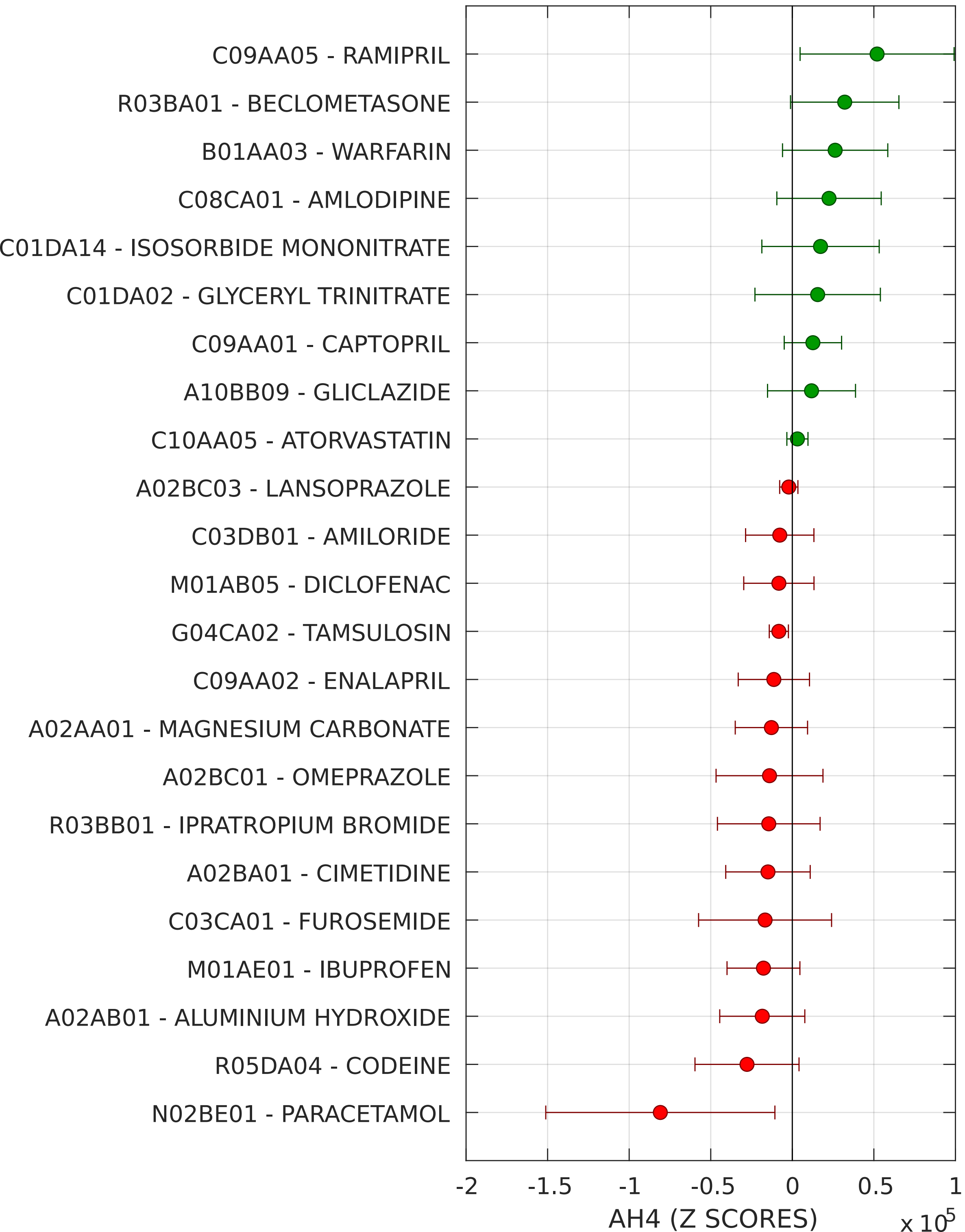

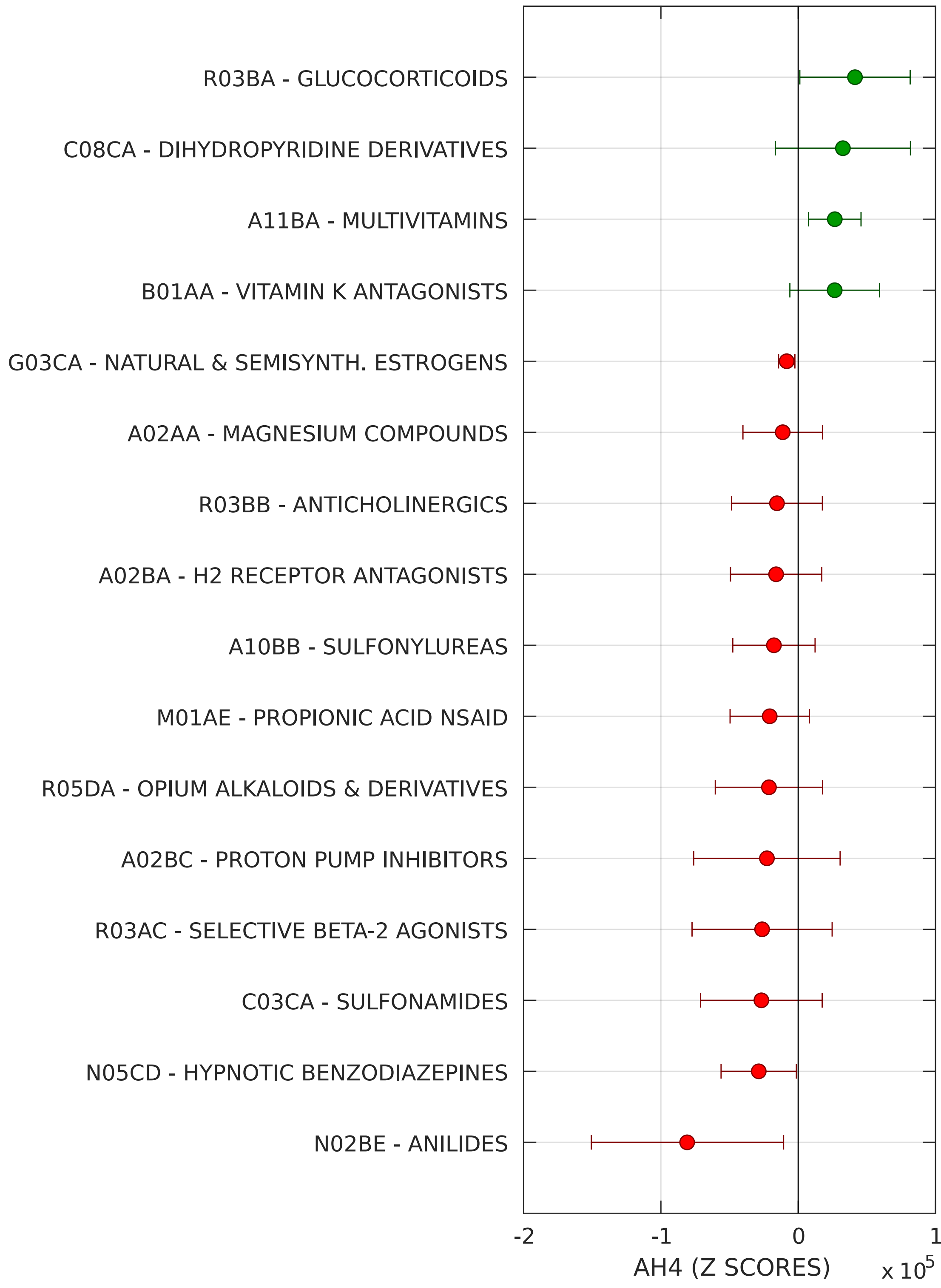

Supplement: Supplementary file 6 — Supplementary Figure 6. Cognitive footprint of medication according to results from Caerphilly Prospective Cohort (CaPS), on the domains of processing speed (choice reaction time ‐CRT‐) and verbal/numerical reasoning (Alice Heim Group Ability Test ‐AH4‐). For each cognitive outcome, the leftward panel presents medications classified according to the last level of the Anatomical Therapeutic Classification (ATC level 5: chemical substances, e.g., ‘ibuprofen’), while the rightward panel groups medications according to the ATC level 4 (pharmacological subgroups, e.g., propionic acid derivatives). Note than while the ATC codes are official, the accompanying terms may have been abbreviated (e.g., ‘tertiary anticholinergics’ for ‘anticholinergics with tertiary amino group’). The cognitive footprint of a medication on a specific cognitive outcome represents the estimated effect of medication use in the UK population (ages 55 to 80), according to the individual effect estimated by modeling CaPS data and assuming UK‐wide prevalence of consumption is the same as in the cohort. Units are Z‐scores of the distribution of the cognitive outcome score (CRT, AH4) across CaPS participants. Error bars represent 95% credible intervals. Only medications with over 50% credibility for a non‐zero effect are presented in the graph (i.e., the 50% credible intervals of the corresponding regression coefficient do not contain zero). Negative values and red color indicate the medication is associated to worse cognitive score, while positive values indicate association to better score. Abbreviations: Non‐ster.: non‐steroidal; antiinflamm: anti‐inflammatory; antirheum: antirheumatic; ACE: angiotensin‐converting enzyme; NSAID: non‐steroidal anti‐inflammatory drugs; inh.: inhibitors; (semi)synth.: (semi)synthetic; ARB: angiotensin II receptor blockers; SSRI: selective serotonin reuptake inhibitors; freq.: frequency; aggreg.: aggregation; excl.: excluding; nsMRI: non‐selective monoamine reuptake inhib [file BRB3-15-e70200-s003.pdf]
