# Supplementary material for: The Cognitive Footprint of Medication Use
Source: Brain Behav. 2025 Jan 19;15(1):e70200. doi: 10.1002/brb3.70200 (PMC11743989; doi:10.1002/brb3.70200)

IMMEDIATE RECALL

RIVERMEAD BEHAVIOURAL MEMORY TEST: IMM-R

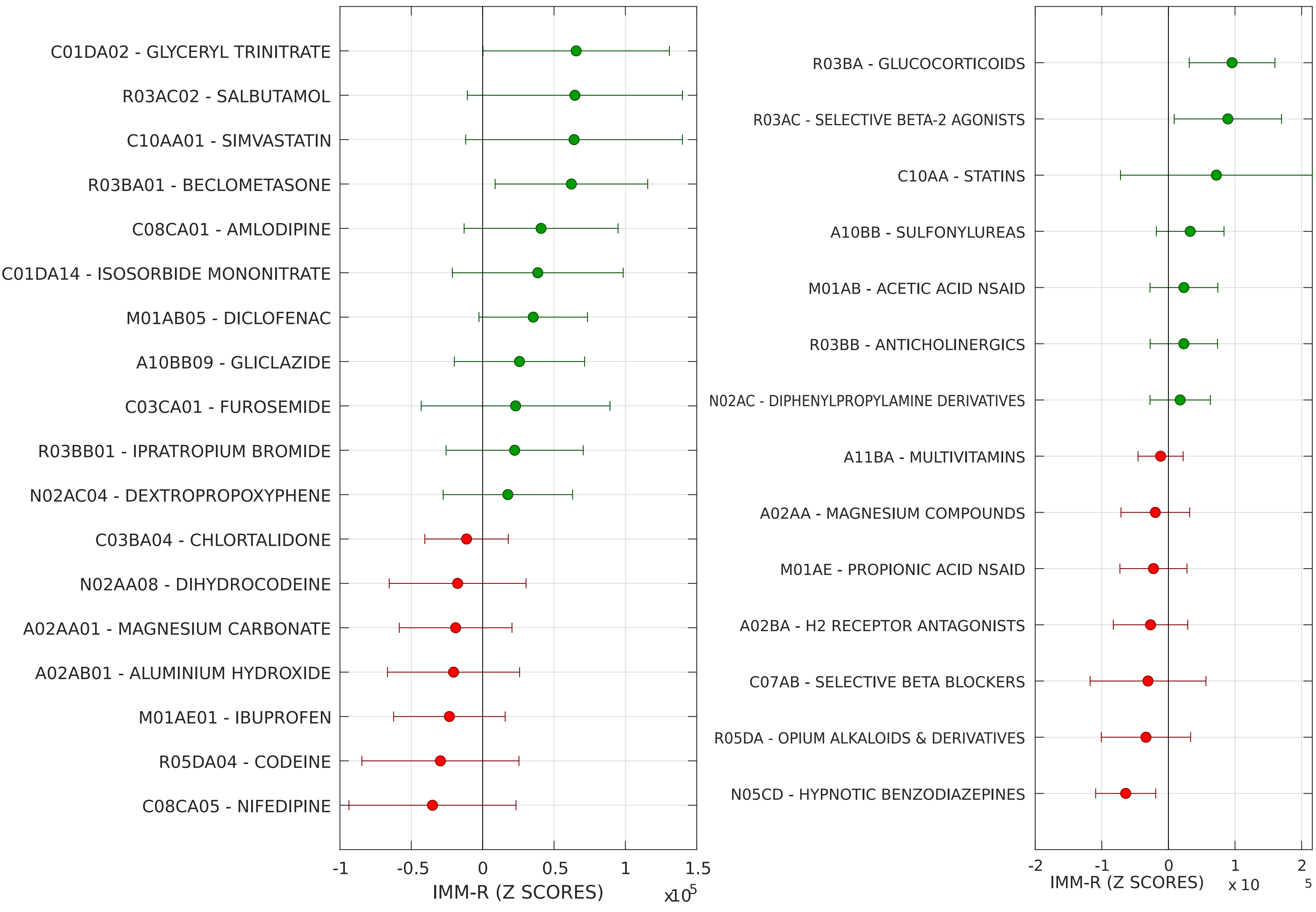

DELAYED RECALL

RIVERMEAD BEHAVIOURAL MEMORY TEST: DEL-R

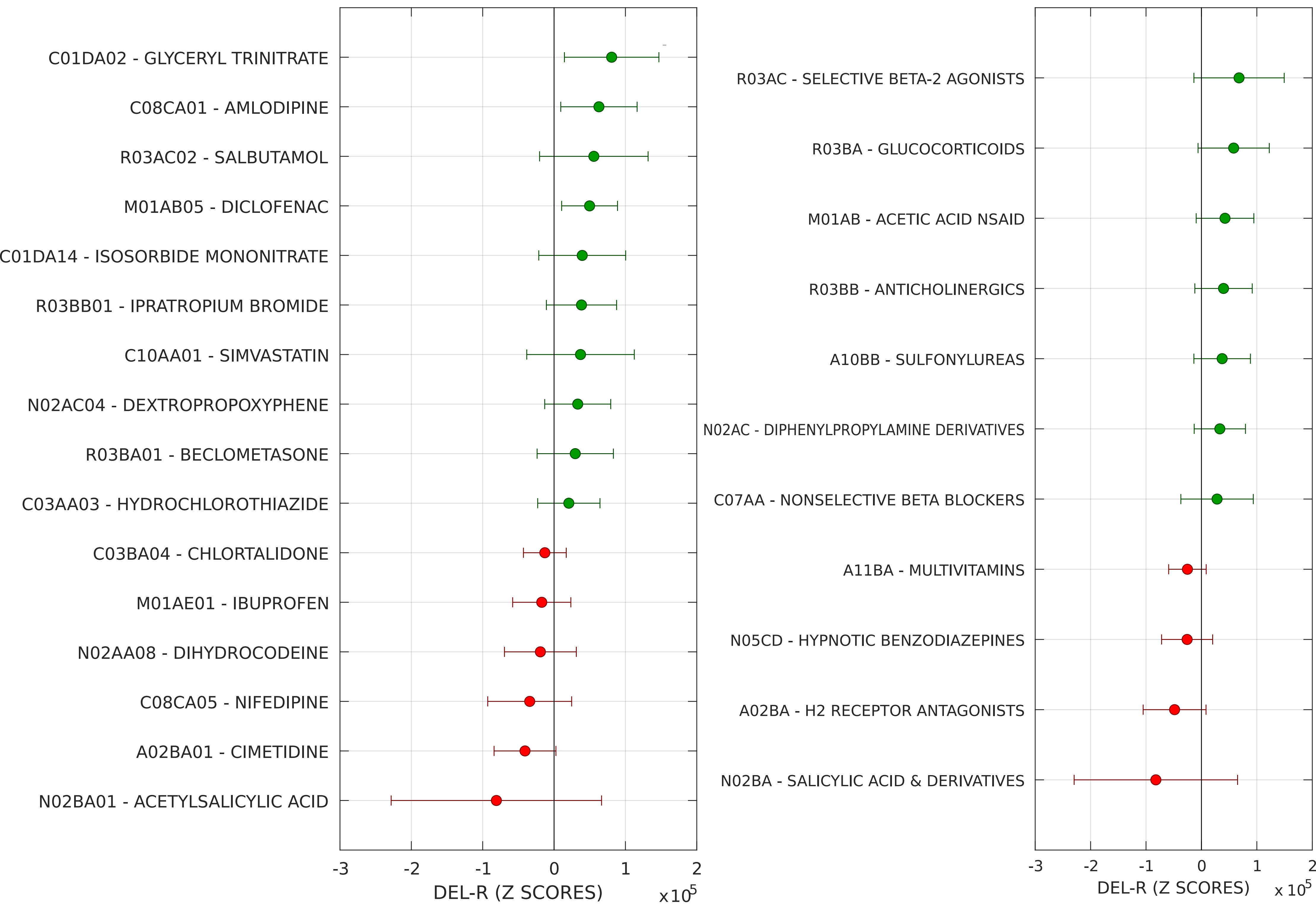

PROMPTED RECALL

RIVERMEAD BEHAVIOURAL MEMORY TEST. PROMPT-R

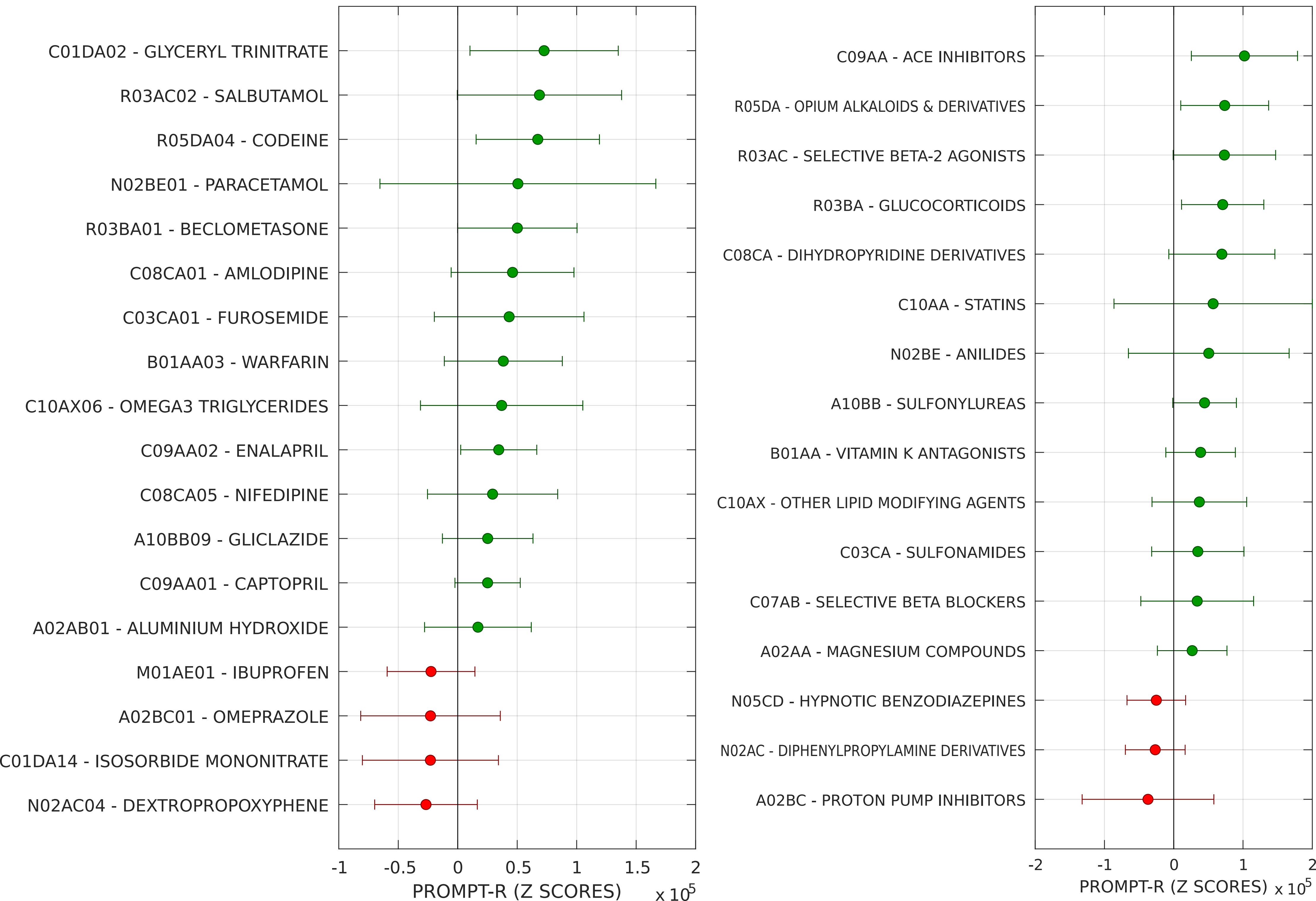

Supplement: Supplementary file 8 — Supplementary Figure 8. Cognitive footprint of medication according to results from Caerphilly Prospective Cohort (CaPS), on the domains of immediate recall (Rivermead Behavioural Memory Test for immediate recall ‐IMM‐R‐), delayed recall (Rivermead Behavioural Memory Test for delayed recall ‐DEL‐R‐) and prompted recall (Rivermead Behavioural Memory Test for prompted recall ‐PROMPT‐R‐). For each cognitive outcome, the leftward panel presents medications classified according to the last level of the Anatomical Therapeutic Classification (ATC level 5: chemical substances, e.g., ‘ibuprofen’), while the rightward panel groups medications according to the ATC level 4 (pharmacological subgroups, e.g., propionic acid derivatives). Note than while the ATC codes are official, the accompanying terms may have been abbreviated (e.g., ‘tertiary anticholinergics’ for ‘anticholinergics with tertiary amino group’). The cognitive footprint of a medication on a specific cognitive outcome represents the estimated effect of medication use in the UK population (ages 55 to 80), according to the individual effect estimated by modeling CaPS data and assuming UK‐wide prevalence of consumption is the same as in the cohort. Units are Z‐scores of the distribution of the cognitive outcome score (IMM‐R, DEL‐R, PROMPT‐R) across CaPS participants. Error bars represent 95% credible intervals. Only medications with over 50% credibility for a non‐zero effect are presented in the graph (i.e., the 50% credible intervals of the corresponding regression coefficient do not contain zero). Negative values and red color indicate the medication is associated to worse cognitive score, while positive values indicate association to better score. Abbreviations: Non‐ster.: non‐steroidal; antiinflamm: anti‐inflammatory; antirheum: antirheumatic; ACE: angiotensin‐converting enzyme; NSAID: non‐steroidal anti‐inflammatory drugs; inh.: inhibitors; (semi)synth.: (semi)synthetic; ARB: angiotensin II receptor blockers; SSR [file BRB3-15-e70200-s010.pdf]
