# Supplementary material for: The Cognitive Footprint of Medication Use
Source: Brain Behav. 2025 Jan 19;15(1):e70200. doi: 10.1002/brb3.70200 (PMC11743989; doi:10.1002/brb3.70200)
Supplement: Supplementary file 10 — Supporting Information [file BRB3-15-e70200-s009.docx]

**Supplementary Material**

**Methods**

For UK Biobank and EPIC Norfolk, modelling was conceived to explore the cross-sectional association between medication use and cognitive performance. In the case of UK Biobank, follow-up assessments were not considered due to the much smaller sample size compared with baseline, and observed stability of the cognitive results. In the EPIC cohort, only the third wave of data collection contained cognitive results. By contrast, the CaPS cohort contained information on medication and cognitive tests for the last 3 waves, without important data loss between assessments, and thus a repeated-measures approach was taken, using the data of waves 3 - 5 as repeated measures. The model output had (beside regression coefficients for covariates, etc) a regression coefficient which represented the main effect of taking one medication in cognition (e.g. paracetamol) and a regression coefficient for the interaction between medication status and wave, which represented how this main effect was modified by progression along waves, considering wave 3 as baseline. Cross-sectional analysis was conducted by Bayesian penalized linear (or logistic) regression with sparse horseshoe-shaped prior, suitable for modelling a large number of potential predictors, while repeated-measures analysis was performed by means of mixed-effects modelling. In the UK Biobank cohort, the sample size of each model depended on availability of data for all the variables. Apart from the occurrence of missing information on model predictors, several cognitive tests (FI, NM, PM) were added or removed halfway through baseline recruitment. Hence, models for tests performed at baseline (FI, NM, RT, PaMa, PM, PCA-Cognition) had a sample size ranging between ~150,000 and over 400,000 participants (except NM with ~46,000). Table 2 shows that summary statistics were similar for all models. In addition to the cognitive assessments, a PCA-Cognition latent variable was calculated as the first component of PCA decomposition of individual cognitive variables - an approach to 'overall cognition'.

Model outputs were employed for estimating the marginal cognitive 'effect' of medication use in each individual, provided that all other variables remained unchanged. In linear models for numeric cognitive variables (or in linear mixed-effects models in CaPS), this was equal to the regression coefficient for the medication. In logistic regression (for PM), the probability of correct response was given by the logistic function applied to each individual, in their actual state and assuming medication was stopped, and averaged within age/sex segments; the expected change in number of correct responses within each segment, due to medication use, could be calculated by binomial distributions.

The overall cognitive footprint of each medication in the UK population according to the results from the UK Biobank cohort was calculated assuming that the prevalence of medication use, stratified by age and sex, was the same as within UK Biobank. The UK population pyramid was obtained from the Office of National Statistics and corresponded to mid-2017. To prevent distortions in stratification, only age/sex segments with more than 1,000 participants in the cohort were considered: thus, although participants' ages at the moment of testing ranged between 37-73 years, only those between 40-70 years were considered. Therefore, overall cognitive footprint pertains only to this age range and not the entire UK population. The same procedure was applied using the results from the other two cohorts, in their corresponding age ranges: 50 – 90 years in EPIC Norfolk and 55 – 80 years in CaPS.

Cognitive variables and domains explored in UK Biobank (Table 2) include: RT (reaction time, related to speed of processing), FI (fluid intelligence score: verbal/numerical reasoning), PaMa (pairs-matching task: short-term visual memory), NM (numeric memory), PM (prospective memory) and PCA-Cognition (first component of PCA decomposition of individual cognitive variables - an approach to 'summary cognition').

Table 2 shows the available N and selected descriptive statistics for the data involved in each model. Out of the 502,492 participants in the UK Biobank cohort, the sample size of each model depended on availability of data for all the variables. Apart from the occurrence of missing information on model predictors, several cognitive tests (FI, NM, PM) were added or removed halfway through baseline recruitment. Hence, models for tests performed at baseline (FI, NM, RT, PaMa, PM, PCA-Cognition) had a sample size ranging between ~150,000 and over 400,000 participants (except NM with ~46,000); at any rate, table 2 shows that summary statistics were largely similar for all models.

Over 54% in the model datasets were female, with a mean age of 56 years. One third were college graduates, while over one fourth had no academic qualification whatsoever; the most frequent household income tier was 31,000 - 52,000 GBP. The most frequent diagnosis (~15%) was dyslipidaemia. More than half reported recent anxious mood and invalidating pain at some point during the last month, while over 22% declared depressed mood during the last 2 weeks. Four medications were taken by over 10% of the sample: paracetamol (~20%), ibuprofen, aspirin and simvastatin.
