# Supplementary material for: The Cognitive Footprint of Medication Use
Source: Brain Behav. 2025 Jan 19;15(1):e70200. doi: 10.1002/brb3.70200 (PMC11743989; doi:10.1002/brb3.70200)
Supplement: Supplementary file 11 — Supporting Information [file BRB3-15-e70200-s006.docx]

**CaPS**

In the CaPS cohort, five medications were taken by over 10% participants in at least one wave: atenolol, omega-3 triglycerides, aspirin, and paracetamol. (Supplementary Figures S5-8)

Similar to UK Biobank and EPIC, paracetamol (anilides) had among the most negative cognitive footprint on several outcomes: choice RT, Alice Heim Group Ability Test (AH4) and measures of global cognition (CAMCOG and MMSE), ranging between -60,000 and -80,000 z-scores (Supplementary Fig S9).

Omega-3 triglycerides had a positive footprint. Unlike UK Biobank and EPIC, M01AX drugs had no relevant positive footprint; however, they were far less used in the older CaPS cohort (e.g. glucosamine was not used by anybody in waves 3 and 4, and only by 2.51% in wave 5).
